# Pharmacological basis of bergapten in gastrointestinal diseases focusing on H^+^/K^+^ ATPase and voltage-gated calcium channel inhibition: A toxicological evaluation on vital organs

**DOI:** 10.3389/fphar.2022.1005154

**Published:** 2022-11-16

**Authors:** Huma Aslam, Arif-ullah Khan, Neelum Gul Qazi, Fawad Ali, Syed Shams ul Hassan, Simona Bungau

**Affiliations:** ^1^ Riphah Institute of Pharmaceutical Sciences, Riphah International University, Islamabad, Pakistan; ^2^ Department of Pharmacy, Kohat University of Science and Technology, Kohat, Pakistan; ^3^ Shanghai Key Laboratory for Molecular Engineering of Chiral Drugs, School of Pharmacy, Shanghai Jiao Tong University, Shanghai, China; ^4^ Department of Natural Product Chemistry, School of Pharmacy, Shanghai Jiao Tong University, Shanghai, China; ^5^ Department of Pharmacy, Faculty of Medicine and Pharmacy, University of Oradea, Oradea, Romania

**Keywords:** bergapten, gastroprotective, anti-ulcer, H/K ATPase, calcium channel blocking, acute toxicity, molecular simulations

## Abstract

**Aim and objectives:** This study aimed to establish a pharmacological basis for evaluating the effects of bergapten (5-methoxypsoralen) in gastrointestinal diseases and assessment of its toxicological profile.

**Methods:** The pharmacokinetic profile was evaluated using the SwissADME tool. AUTODOCK and PyRx were used for evaluating the binding affinities. The obtained results were further investigated for a post-dock analysis using Discovery Studio Visualizer 2016. The Desmond software package was used to conduct molecular dynamic simulations of best bound poses. Bergapten was further investigated for antidiarrheal, anti-secretory, charcoal meal transit time, anti-ulcer, anti-*H. pylori* activity.

**Results:** Bergapten at a dose of 50, 100, and 200 mg/kg was proved effective in reducing diarrheal secretions, intestinal secretions, and distance moved by charcoal meal. Bergapten at the aforementioned doses acts as a gastroprotective agent in the ethanol-induced ulcer model that can be attributed to its effectiveness against *H. pylori.* Bergapten shows concentration-dependent relaxation of both spontaneous and K^+^ (80 mM)-induced contractions in the isolated rabbit jejunum model; the Ca^2+^ concentration–response curves (CRCs) were shifted to the right showing potentiating effect similar to papaverine. For molecular investigation, the H^+^/K^+^ ATPase inhibitory assay indicated inhibition of the pump comparable to omeprazole. Oxidative stress markers GST, GSH, and catalase showed increased expression, whereas the expression of LPO (lipid peroxidation) was reduced. Histopathological examination indicated marked improvement in cellular morphology. ELISA and western blot confirmed the reduction in inflammatory mediator expression. RT-PCR reduced the mRNA expression level of H^+^/K^+^ ATPase, confirming inhibition of the pump. The toxicological profile of bergapten was evaluated by an acute toxicity assay and evaluated for behavioral analysis, and the vital organs were used to analyze biochemical, hematological, and histopathological examination.

**Conclusion:** Bergapten at the tested doses proved to be an antioxidant, anti-inflammatory, anti-ulcer, and antidiarrheal agent and relatively safe in acute toxicity assay.

## 1 Introduction

More than 8 million people worldwide die from gastrointestinal conditions each year, which include gastrointestinal malignancies, gastroesophageal reflux disease, *Helicobacter pylori* infection, peptic ulcer disease, inflammatory bowel disease, celiac disease, and functional gastrointestinal dysfunction (Milivojevic and Milosavljevic, 2020). The prevalence of GI diseases has an increasing trend owing to the environmental factors including lifestyle, hygiene, and infection exposures (Ohama et al., 2007). Multiple GI disorders including inflammatory bowel disease, gastric ulcers, *Helicobacter pylori (H. pylori)* infections, colorectal cancers, diarrhea, constipation, and gastroesophageal reflux disease are most prevailing globally. According to surveys performed by the World Gastroenterology Organization, *H. pylori* infection is most common in the adult population and has shown median prevalence in about 50% of the world population. WHO data for recent global disease burden reports diarrheal diseases to be among the top 10 causes of death globally. Diarrheal diseases account for approximately 1.4 million deaths worldwide in 2016. Colorectal cancer is the fourth leading cause of cancer related death, accounting for over 4,900 deaths in 2014 (Jones et al., 2017). Although treatment options are available, the consequences and mortalities are still a problem because of poor treatment adherence of the patients usually due to multidrug dosing regimens.

The gastrointestinal tract shows contractile motions along with secretions throughout the tract; hence, any alteration in these may cause increased or decreased GI motility and contractions, which may further cause certain conditions such as diarrhea, constipation, abdominal cramping, and indigestion. Intestinal motility, on the other hand, may be affected by underlying disease conditions or certain medications (Hasler et al., 2010). While some ailments such as ulcers are associated with the imbalance in protective and aggravating factors in GIT, many bacterial infections also cause this imbalance and contribute toward breaching of the mucus lining. Therefore, strenuous antibiotic therapy is often recommended that causes patient non-compliance (Nyssen et al., 2021). Moreover psychological health, overall health condition, and dietary factors that influence intestinal content can also affect sensory signaling and, hence, motility in the gastrointestinal tract (Hasler et al., 2010). The imbalance in aggressive and protective aspects leads to increase in acid secretions from gastroparietal cells causing ulcers. Other aspects such as gastrin, histamine, muscarinic, and prostaglandin-E2 signaling may also affect H^+^/K^+^ ATPase, which is involved in the release of H^+^ ions causing an acidic environment (Mishra, 2016). Therefore, H^+^/K^+^ ATPase is a promising target while treating ulcers. Calcium channel blockers, on the other hand, also proved therapeutically beneficial in ulcers and other GI motility ailments by increasing microcirculation, gastric function, and GI motility by affecting signaling pathways (Fomina, 2020; Khakimov, 2021). Medicinal science so far has not been able to produce any efficient remedial drug in contrast to gastrointestinal disorders which can completely eradicate the disease, and current treatment options provide temporary relief of symptoms (Irvine et al., 2006).

Secondary metabolites from nature, predominantly plants, are still a research hotspot because of their promising novel scaffolds against chronic diseases (Muhammad et al., 2021a; Memariani et al., 2021; Majid et al., 2022). Medicinal plants are used traditionally as a source of complementary and alternative medicine in gastrointestinal disorders (Bahmani et al., 2014). Medicinal plants and their isolated compounds account for approximately 30% of all pharmaceuticals, becoming globally important in recent years (Khan et al., 2019; Muhammad et al., 2021b; Ul Hassan et al., 2021). Additionally, herbal treatments have demonstrated good long-term economic potential to cure problems of the gastrointestinal system. Bergapten (5-methoxypsoralen) is a furanocoumarin that is commonly found in lemon, grapefruit, fig, Cnidii Fructus, and many medicinal plants (Liang et al., 2021). It acts as an antioxidant, anti-insect, analgesic, anti-inflammatory, anticoagulant, and anticancer agent (Yu et al., 2016). Plants from the Apiaceae and Rutaceae family contain furanocoumarins as major phytoconstituents and have proved their potential in several GI disorders. Medicinal plants from these families contain bergapten as one of main constituents, therefore leading to basis for selection of this compound to evaluate scientifically for gastrointestinal disorders (Abbaskhan et al., 2011; Vera Saltos et al., 2013). Recent studies proved bergapten to have antidiabetic properties, and due to its ability to cross the blood–brain barrier, it is expected to be a useful therapeutic agent in certain brain ailments such as diabetes-associated Alzheimer’s, epilepsy, and depression (Liang et al., 2021). Bergapten was also found to have good binding affinity and potential to act as protease inhibitor against SARS-CoV-2 during *in silico* analysis (Chidambaram et al., 2020). Through *in vivo* and *in silico* evaluation of bergapten, it could be formulated into a suitable dosage form. In this study, we have initially tested bergapten through *in silico* studies using a docking analysis and molecular dynamic simulation studies against selected favorable target proteins involved in GI ailments.

The aim of this study was to assess the potential capability of bergapten as a therapeutic agent in several GI diseases. Assuming that bergapten modulates the expression of proteins in the GI tract, it was evaluated for its effectiveness including anti-spasmodic, anti-motility, gastrointestinal transient time, anti-ulcer potential, calcium channel blocking, anti-*H. pylori,* H^+^/K^+^ ATPase activity, histopathological examination, immunohistochemistry, ELISA, western blotting assay, RT-PCR, and computational analysis including molecular simulation studies. Importantly, the gastroprotective role of bergapten in gastric ulcers and its regulation at the molecular and organ/tissue damage level is examined focusing on H^+^/K^+^ ATPase and calcium channel blocking activity. *In silico* and proteomic approaches aid in understanding the biochemical mechanism and, as a result, may untangle the complicated signaling network that affects cellular function such as apoptosis and inflammation.

## 2 Materials and methods

### 2.1 Chemicals

Castor oil was bought from a company in Pakistan called Karachi Chemical Industries. All the other ingredients came from Sigma Chemicals Co. in St. Louis, Missouri, United States; they were as follows: acetylcholine, activated charcoal, bergapten, ethanol, loperamide, verapamil, papaverine, atropine, and omeprazole.

### 2.2 Animals

The studies were conducted according to the “Principles of Laboratory Animal Care” and the rules of the Research and Ethics Committee of the Riphah Institute of Pharmaceutical Sciences (RIPS) (Ref. no. REC/RIPS/2019/021). Sprague Dawley rats (190–240 g) of either gender, rabbits (1.0–2.0 kg), and Balb/c mice were used in the study (20–25 g). At an animal house at RIPS, Islamabad, all the animals were kept in a controlled environment.

### 2.3 Computational studies

Reference pharmaceuticals’ three-dimensional structures were created in 2016 using the Discovery Studio Visualizer by adding polar hydrogen atoms; then, they were turned into a PDB file. Papaverine, phenylephrine, pirenzepine, atropine, domperidone, calmidazolium, verapamil, and ranitidine were some of the most commonly used drugs for docking. The RCSB Protein Data Bank was used to choose and get targets related to gut physiology. Calmodulin, dopaminergic D2, adrenergic 1 receptor, histaminergic H2, muscarinic M1, mu-opioid, phosphodiesterase enzyme, and H^+^/K^+^ ATPase with their PDB IDs 1CTR, 6CM4, P35348, P25021, 5CXV, 6DDE, 6DDE, and 1T3S, respectively, were chosen. DSV was used to clean up the structures. PyRx was used to dock molecules (Faheem et al., 2021; Shams ul Hassan et al., 2021).

### 2.4 SwissADME

SwissADME is an online method to access the ADME (Absorption, Distribution, Metabolism, and Excretion), that is, the pharmacokinetic profile including drug likeliness, physicochemical, lipophilicity, solubility properties, and medicinal chemistry (Faheem et al., 2021; Shams ul Hassan et al., 2022).

### 2.5 Molecular dynamics simulation

The probable binding interactions of the target protein H^+^/K^+^ ATPase and voltage-dependent calcium channel (VDCC) were predicted using a docking analysis. Later, molecular docking simulation of H^+^/K^+^ ATPase–bergapten and VDCC–bergapten complexes were conducted using the Desmond software package of SCHRÖDINGER 2020-3 version. The aforementioned complexes were placed in a molecular simulation in an orthorhombic box that was automatically created and supplemented by 55,180 and 13,403 for the complexes H^+^/K ^+^ATPase-bergapten and VDCC-bergapten, respectively. Utilizing liquid simulations with optimized potential, the model is further enhanced (OPLS5). NaCl was added to make the solution isotonic and to neutralize the system’s complexes (Na = 52.06 mM and Cl = 50.74 mM). Counter ions were injected to provide an electrically stable environment. Temperature and pressure were maintained at 300 K using a Nose–Hoover thermostat and 1.01325 bars using Martyna–Tobias–Klienbarostate, respectively. The MD simulation total time was 100 ns. The Eswald method was used for calculating electrostatic interactions. The simulation interaction diagram tool in the Desmond package was used to evaluate interaction behavior between the ligand and protein. Structural optimization of ligand was performed using the density functional theory method. The Desmond software gives results in the form of RMSD for the protein and RMSF for the residue. These are then used to evaluate the results further (Ali et al., 2022; Hassan et al., 2022).

### 2.6 Castor oil-induced diarrhea

This protocol was conducted as previously described by Ansari et al. (2019). Balb/c mice were randomly allocated to five groups for bergapten and fasted for 24 h (08:00–08:00), before experimentation. Animals were kept in separate cages and distributed in equal groups lining the floor of cage with absorbent paper. After an hour, negative control mice were administered 10 ml/kg of castor oil orally (P.O.). Mice in the positive control group were given loperamide hydrochloride at a dose of 2 mg/kg of body weight. Following the administration of bergapten at dosages of 50 and 100 mg/kg, diarrhea was induced by administering castor oil (10 ml/kg) orally. Animals were placed in separate cages lined with white paper. Each animal was monitored for a whole period of 4 hours in order to ascertain the time of the commencement of diarrhea and the presence or absence of diarrheal droppings. The percentage of diarrheal protection was evaluated according to the Sisay et al. (2017).

### 2.7 Assessment of intestinal fluid accumulation

Intestinal fluid accumulation was determined using the method described by Ansari et al. (2019). An enteropooling assay was used to evaluate the accumulation of intestinal fluid. Mice fasting for 24 h (08:00–08:00) were removed and kept into six designated cages, each holding five mice. Group I and II were administered with normal saline (10 ml/kg) and castor oil (10 ml/kg, p.o.), respectively. In addition, 1 h prior to induction with castor oil (10 ml/kg, p.o.), bergapten at doses of 50, 100, and 200 mg/kg was given intraperitoneally to Group III, IV, and V, respectively. Standard drug atropine at dose 10 mg/kg was given to VI group. Mice were sacrificed after 30 min, intestine was removed and weighed. The results were articulated as (Pi/Pm) x 1000, where Pi is the weight (g) of the intestine and Pm is the weight of the animal.

### 2.8 Charcoal meal transit time

The inhibitory activity of the propulsion of a charcoal meal in rats was estimated utilizing bergapten. The rats were fasted for 24 h, but had a free access to water. The test groups received the doses of bergapten at 50, 100, and 200 mg/kg body weight doses; the positive control group received atropine sulfate (0.1 mg/kg, i.p.), while the negative control group received normal saline (10 ml/kg, p.o.). All groups were treated with a period marker 25 mg/kg (10% activated charcoal suspension in 5% gum acacia) 1 hour after pretreatment. After all treatments, animals were sacrificed. The small intestine was excised, and then the distance traveled by charcoal meal 1 ml of marker through the organ was expressed as a percentage of the length of the small intestine according to the following expression (Ansari et al., 2021):

Peristaltic index(PI%)=(Distance moved by charcoal meal/total length of intestine)(cm)×100.

For further evaluation of % inhibition, the peristaltic index is used.

% inhibition = (PIC-PIT)/PIC X 100,

where PIC = peristaltic index of control and PIT = peristaltic index of test group.

### 2.9 Effect of bergapten on the motility of isolated tissue preparations

Before experimentation, the rabbits were fasted for 24 h (08:00–08:00), although they were given free access to water. It was around 10–12 cm after cervical dislocation that the jejunum was separated and cleansed with Tyrode’s solution. Before administering the medication, 2 cm of jejunum was suspended in Tyrode’s solution. Later, 30 min of acclimatization in a controlled environment with 95% oxygen and 5% carbon dioxide (carbogen) supply enabled the jejunal section to adapt to the new environment. They were recorded by connecting the force displacement transducer (model FDT-33) through the bridge amplifier and power Lab 4/25 data recording equipment to a computer running the lab data acquisition and analysis application on the computer. Each preparation was stabilized with a concentration of ACh (0.3 M) (AD Instrument, Sydney Australia). The percent change in jejunal contractions was examined at various levels of bergapten concentration (Rehman et al., 2011; Rehman et al., 2012). High K^+^(80 mM) depolarizes the preparations for smooth muscle contractions by opening voltage-dependent Ca^2+^ channels, which results in extracellular Ca^2+^ influx and has a contractile effect. Substances that inhibit the high K^+^-induced contraction are thought to block Ca^2+^ influx through L-type Ca^2+^ channels. After the generated contraction had initially plateaued (often within 7–10 min), test dosages were increased cumulatively to produce concentration-dependent inhibitory responses. The tissue was allowed to stabilize in regular Tyrode’s solution before being switched to Ca^2+^ free Tyrode’s solution containing EDTA (0.1 mM) for 30 min. This was carried out to validate the test substance’s Ca^2+^ antagonist effect. Additionally, tissue was put in a Tyrode’s solution that contained K^+^ but no Ca^2+^. The control concentration–response curves (CRCs) of Ca^2+^ were produced after a 30-min incubation period. The tissue was pretreated with test dose for 1 h after it was established that the control Ca^2+^ CRCs were super-imposable (often after two cycles). To detect the Ca^2+^ antagonist effect, the CRCs of Ca^2+^ were reconstructed in the presence of various concentrations of the test substance (Khan and Gilani, 2009).

### 2.10 Anti-*Helicobactor pylori* activity

The resistant strains of *H. pylori* clinical isolates were obtained from PINSTECH (Pakistan Institute of Nuclear Science and Technology, Islamabad, Pakistan). Identification assays including morphology, gram staining, urease test, and catalase tests were performed for checking microaerophilic growth (at 37°C) (Ullah et al., 2019). The disc diffusion method was used to analyze an antibacterial effect of bergapten, measuring the zone of inhibition in mm using 5 mg of bergapten per disc. The obtained *H. pylori* clinical isolate SJ 2013 10^8^ cfu/ml was resistant to antibiotics such as amoxicillin, clarithromycin, and metronidazole. These isolates were inoculated on Columbia blood agar (CM 0331B, Oxoid, UK) enriched with 5% defibrinated sheep blood. Discs impregnated were applied and incubated at 37°C for 72 h under microaerophilic conditions using the Campylobacter gas generation kit (BR 0056A, Oxoid, United Kingdom). After completion of incubation of 72 h, the diameter around each disc was measured to access the zones of inhibition. The microdilution method with brain heart infusion (BHI) broth was used to access minimum inhibitory concentration (MIC) for bergapten. Serial dilution by two folds in BHI broth with serum was used, and final concentrations of extracts were 0.625–5.0 mg/ml. The concentration at which with no visual growth or turbidity was observed was considered as MIC.

### 2.11 Ethanol-induced ulcer assay

The rats of either sex that weighed between 250 and 280 g were randomly divided into groups and kept for a 24-h fast. As a negative control, Group 1 was given 10 ml/kg of normal saline and Group 2 received omeprazole 20 mg/kg as a standard drug. Groups 3, 4, and 5 were given 50, 100, and 200 mg/kg of bergapten, respectively. In order to induce stomach ulcers in all of the animals, 1 ml/100 g of absolute ethanol (100%) was administered intraperitoneally after an hour of treatment. Animals were sacrificed 1 hour after consuming ethanol. Stomachs were removed and washed with normal saline, and each lesion was calculated according to the protocol previous studied by Noor et al. (2017) and Li et al. (2022). For further proteomic analysis, the gastric tissues were reserved in a bio-freezer (−80°C).

### 2.12 H^+^/K^+^ ATPase inhibitory assay

The inhibitory effect of bergapten on rat gastric H^+^/K^+^ ATPase inhibitory effect was accessed using a commercially available colorimetric H^+^/K^+^ ATPase activity assay screening kit (catalog No. E-BC-K122-S, Elabscience USA). A bio-freezer (−80°C) was used to retain the treated stomach tissues before they were homogenised at 15,000 using the Silent Crusher M. (Heidolph). After centrifugation at 3500 rpm for 10 min, the homogenate was finally separated. We used a spectrophotometric assay (660 nm) to check for the release of inorganic phosphate from the collected supernatant after it had been collected (He et al., 2018). In addition, 1 µmol of inorganic phosphorus released by ATP hydrolysis by ATPase of 1 mg of tissue protein per hour is defined as 1 ATPase activity and expressed as µmol Pi/mg prot/hour.

### 2.13 Determination of oxidative stress markers

The supernatant collected after homogenization and centrifugation, respectively, of isolated rat gastric tissue at 1500 rpm for 30 min, the level of glutathione (GSH), glutathione-S-transferase (GST), catalase, and lipid peroxidation (LPO) was examined in the supernatant. The oxidation of GSH and DTNP (2,2′-Dithiobis (5-nitropyridine)) produces 2-nitro-5-thiobenzoic acid, which is a yellow end product. To access the levels of GSH after oxidizing GSH and DTNP, a microplate reader was used to measure absorbance at 412 nm and expressed as μmole/mg of protein. Formation of CDNB (2,4-dinitrochlorobenzene) conjugate is used to access GST levels. Conjugate formation is measured by absorbance at 340 nm using the extinction coefficient of the product formed and is expressed as μmoles of CDNB conjugate/min/mg of proteins. Catalase activity is measured in moles H2O2/min/mg of protein by measuring the degradation of H_2_O_2_ and using microplate reader to measure absorbance at 240 nm. LPO (lipid peroxidation) values are measured in TBARS (thiobarbituric acid reactive substances) nmoles/min/mg protein. The end product of LPO is malondialdehyde, which is measured using a microplate reader and absorbance at 532 nm (Qazi et al., 2022). For SOD (superoxide dismutase), an assay was performed according to Dengiz et al. (2007). Superoxide radicals generated by xanthine and xanthine oxidase and reaction with nitro blue tetrazolium produces formazan dye. Superoxide dismutase activity was estimated by inhibition of the aforementioned reaction and inhibition determined at 560 nm and expressed as millimole per minute per milligram tissue (mmol/min/mg/tissue).

### 2.14 Morphology analysis, histopathology, and H&E staining

A total of five rats were used in each group for morphological examination. For fixation of the stomach tissues, 4% paraformaldehyde is used, and until further analysis, tissues are implanted and fixed in paraffin. The fixed tissues were then sectioned using a rotary microtome at 5 μm. These were stained with hematoxylin and eosin (H&E) and observed under an optical microscope, and photos were taken according to Ansari et al. (2021) and Liu et al. (2022a).

### 2.15 Proteomics of stomach tissues by using enzyme-linked immunosorbent assay

Tumor necrosis factor alpha (TNF-α), prostaglandin-E_2_ (PGE_2_), and interleukin-8 (IL-8) were detected according to the manufacturer’s instructions. The stomach tissues (*n* = 3) stored in the bio-freezer (−80°C) were homogenize at 15 rpm × 1000 using Silent Crusher M (Heidolph) and later centrifuged (at 1350 × g for 1 h) for collection of supernatants. It was then analyzed for quantification of TNF-α (Catalog No: E-EL-R0019), PGE2 (Catalog No: E-EL-0034), and IL-8 (Catalog No: EKF57830) using an Elabscience Rat ELISA kit (Liu Z. et al., 2022b).

### 2.16 Western blot analysis

To determine the expression of inflammatory mediators involved in progression of ulcer, a western blot analysis was used. Sample proteins (*n* = 3) from each experimental group were prepared with the addition of laemmli buffer, vortexed, and incubated at a temperature of 96°C for 10 min. These were then placed in ice for 10 min after incubation and again vortexed. After preparation of gel protein samples were loaded in polyacrylamide gels separated by SDS/PAGE. The negative charge on SDS is used for protein denaturation, and polyacrylamide allows the movement of protein at altered speed based on their charge. After separation of proteins, they were shifted to polyvinylidene fluoride (PVDF) membrane for the trans blot process. A 5% skimmed milk solution was prepared in TBST (Tris-buffered saline with Tween) buffer and used for washing and blocking membranes for non-specific binding. Primary antibodies including phosphorylated nuclear factor kappa β (SC-271908 Santa Cruz Biotechnology, Dallas, TX, United States), tumor necrosis factor α (SC-52B83 Santa Cruz Biotechnology, Dallas, TX, United States), and COX-2 (SC-514489 Santa Cruz Biotechnology, Dallas, TX, United States) were applied on membranes and were incubated for 16 h at 4°C. Membranes were washed in TBST buffer for 10 min, and anti-mouse secondary antibody was applied for 3–4 h at room temperature. By interacting and binding with the primary antibody, the secondary antibody detects target protein. The PVDF membrane was then again washed in TBST, and proteins on the PVDF membrane were transferred to X-ray sheet. Band density was measured and standardized to β-actin (Noman et al., 2022).

### 2.17 Reverse transcription-polymerase chain reaction analysis

Following the manufacturer’s instructions, the TRIzol technique was used to extract total ribonucleic acid (RNA) after homogenization of gastric tissues (*n* = 3), and cDNA was synthesized by reverse transcriptase enzyme using 1–2 µg of total RNA. cDNA was then amplified by real-time PCR using a thermocycler. Beta-actin expression levels were used to normalize mRNA expression. For real-time quantitative PCR, the relative gene expression was determined by using the 2^^ΔΔ−CT^ method (Ali et al., 2022). Primers sequences for β-actin and H^+^/K^+^ ATPase are as follows:

**Table:** 

Primers	Tm	Sequence (5′–3′)
Rat_BetaActin_Forward	60.5°C	CCCGCGAGTACAACCTTCT
Rat_BetaActin_Reverse	59.5°C	CGTCATCCATGGCGAACT
H^+^/K^+^ ATPase forward	52.3°C	TATGAATTGTACTCAGTGGA
H^+^/K^+^ ATPase reverse	53.9°C	TGGTCTGGTACTTCTGCT

### 2.18 Toxicity studies

In total, two groups of female rats (*n* = 5) were obtained. One was control group and the other treatment group. In this study, we used non-pregnant, nulliparous female rats to test acute toxicity of bergapten. One animal at a time was fasted overnight and administered with 2000 mg/kg of bergapten in accordance with OECD standards 425 (Saleem et al., 2017; Song and Wu., 2022). The rat was observed for 24 h, and if it survived, the other rats in the therapy group were given the same treatment; writhing, salivation, twitching, convulsions, loss of fur, and stress were among the symptoms seen. These symptoms were observed for 48 h, followed by 14 days of daily monitoring. As soon as the animals had been anaesthetized and their essential organs removed, blood was drawn from their hearts by cardiac puncture. For example, these blood samples were used to determine the moist weight of their organs and to compile antioxidant profiles and tests to determine how well their livers and kidneys functioned.

#### 2.18.1 Biochemical analysis of the toxicity study

Biochemical markers such as lipid profile (cholesterol, triglyceride, low-density lipoprotein (LDL), very-low-density lipoprotein (VLDL), and high-density lipoprotein (HDL)), renal function tests (urea, uric acid, and creatinine), liver function tests (alanine aminotransferase (ALT), bilirubin, aspartate aminotransferase (AST), and alkaline phosphate (ALP)), and total protein were estimated using a Randox kit.

#### 2.18.2 Hematological analysis of the toxicity study

EDTA tubes were used to collect blood samples from control and treated animal. HumaLyzer was used to access the hematological parameters such as complete blood count (CBC), hemoglobin (Hb) levels, red blood cell count, white blood cell count, platelet count, packed cell volume (PCV), mean corpuscular volume (MCV), mean corpuscular hemoglobin (MCH), neutrophils, lymphocytes, monocytes, and eosinophils (Lei et al., 2022).

#### 2.18.3 Histopathological analysis of the toxicity study

The animals subjected to toxicity study were sacrificed and fixed in 10% formalin and embedded in paraffin wax. H&E (hematoxylin and eosin) staining was used to stain on paraffin embedded organ sections and slides were prepared and observed under a light microscope for further investigation.

### 2.19 Statistical analysis

Data were expressed as mean ± SEM (*n* = 5) and median effective concentrations (EC_50_) having 95% confidence intervals. A statistical analysis of the results was carried out using one-way ANOVA followed by *post hoc* Tukey’s test. Non-linear regression using GraphPad program (GraphPad, SanDiego, CA, United States) was used to analyze the concentration–response curves.

## 3 Results

### 3.1 Docking study

Energy levels and H-bonds determine docking assessment. Bergapten’s binding affinities to protein receptors vary. Supplementary Table S1 provides atomic energy values (kcal/mol) and residues implicated in H-bonding, pi-pi bonding, and other hydrophobic interactions of bergapten and reference. Supplementary Figures S1–S11 illustrate bergapten’s 2D interactions with typical medicines.

#### 3.1.1 Targets information

The total residues, protein chains, number of atoms, charge, and heavy atoms on the protein target H^+/^K^+^ ATPase (4UX2) and voltage-gated L-type calcium channel (1T3S) assessed during simulation are presented in Supplementary Figures S12A, S13A. The RMSF in Å A° was 2.5 for 4UX2 and 5.5 for 1T3S indicated in Supplementary Figures S12B, S13B. In Supplementary Figures S12C, S13C, for 4UX2 and 1T3S, red represents alpha helices and blue beta strands.

#### 3.1.2 Ligand information

In Supplementary Figure S14A, the root mean square deviation (RMSD), extendedness of the ligand as measured by the radius of gyration (rGyr), number of internal H-bonds within the ligand, molecular surface area (MolSA) calculation with 1.4 A° probe radius, solvent accessible surface area of the ligand (SASA), and polar surface area (PSA), which is contributed only by the oxygen and nitrogen atoms Supplementary Figure S14B shows Bergapten’s structure, atom count, atomic mass, charge, molecular formula, fragments, and rotatable bonds.

### 3.2 SwissADME

Pharmacokinetic profile including drug likeliness, physicochemical, lipophilicity, and solubility properties are described in Supplementary Table S2.

### 3.3 Molecular dynamic simulations

**FIGURE 1:**
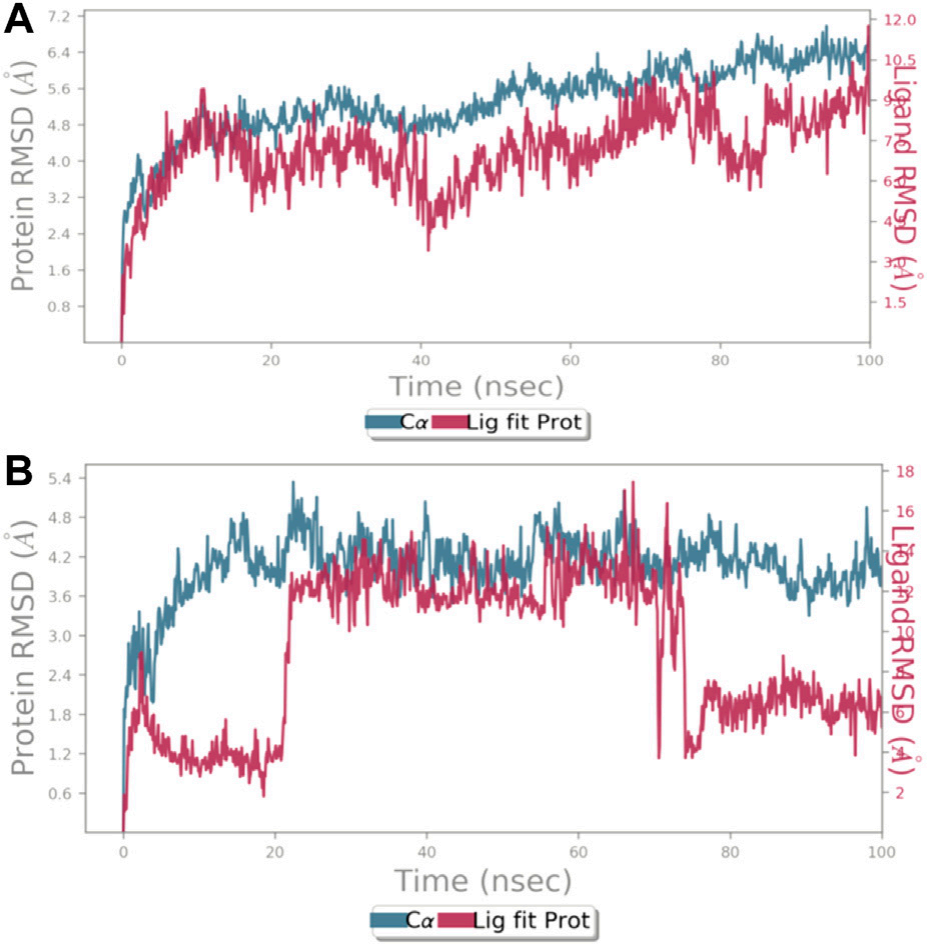
**(A)** represents root mean square deviation (RMSD) for protein 4UX2 shown in blue and RMSD of bergapten shown in red. **(B)** RMSD for protein 1T3S shown in blue and RMSD of bergapten shown in red using the Desmond software package.

**FIGURE 2:**
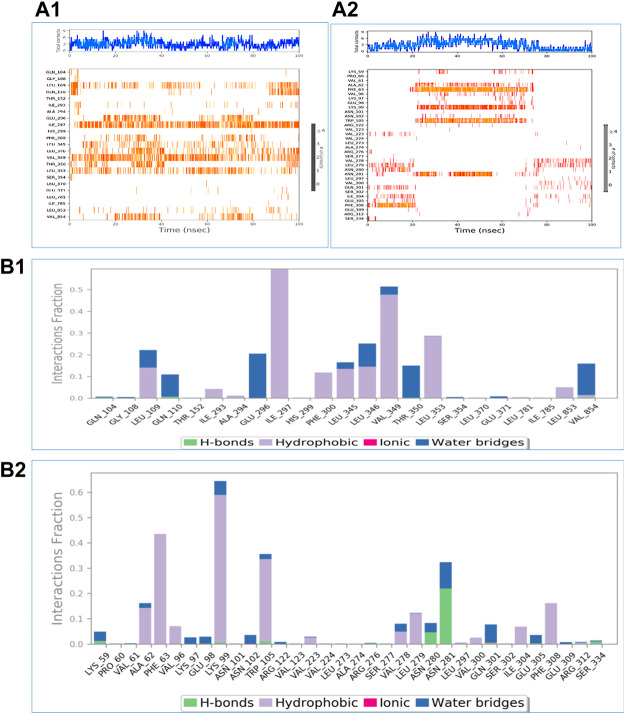
A time-based depiction of the multiple interactions and contacts including hydrogen, hydrophobic, ionic bonds, and water bridges between bergapten vs. 4UX2 and 1T3S are presented in **(A1,A2)**, respectively. The total number of specific interactions between the protein and the ligand is shown in the top panel. The interacting residues with the ligand are shown in the bottom panel. Some residues form more than one specific contact with the ligand, and these are indicated by deeper hue of Orange, according to the scale to the right of the plot. **(B1,B2)** exhibit protein interactions between 4UX2 and bergapten and 1T3S and bergapten, respectively. These interactions are categorized as hydrogen bonds, hydrophobic, ionic, and water bridges.

Using the Desmond software package, molecular dynamic simulation was performed. The physiological environment was provided to the complexes and were run for 100 ns. RMSF of the atomic positions for bergapten on 4UX2 is shown in Supplementary Figures S15A and bergapten on IT3S is shown in Supplementary Figure S15B. Root mean square deviation (RMSD) of interaction between 4UX2 and IT3S with bergapten is shown in Figures 1A,B, respectively. Small deviation in RMSD shows stable binding. Interactions of bergapten with different amino acid residues on 4UX2 and 1T3S are displayed in Figures 2A1,A2, respectively. Some residues form more than one specific contact with the ligand, which is depicted as darker shade of orange. Other interactions including H-bonds, hydrophobic, ionic, and water bridges between bergapten vs. 4UX2 and 1T3S are represented in Figures 2B1,B2, respectively.

### 3.4 Effect of bergapten on castor oil-induced diarrhea

In contrast to the saline-treated negative control group, which showed no protection against castor oil-induced diarrhea, bergapten had a dose-dependent protective effect (50–100 mg/kg) in mice. To prevent diarrhea, bergapten offered 40% protection at 50 mg/kg, and 100% protection at 100 mg/kg (*p* < 0.05 vs. the saline group). Loperamide, a well-known antidiarrheal, prevented diarrhea in positive control shown in Table 1 (*p* < 0.001 vs. castor oil).

**TABLE 1:** Effect of bergapten and loperamide against castor oil-induced diarrhea in mice.

Treatment (mg/kg)	No. of wet feces	Total no. of feces	Average weight of wet feces (gm)	Average weight of total feces (gm)	% inhibition of defecation	% WWFO	% WTFO
Saline (10 ml/kg) + castor oil (10 ml/kg)	8.0 ± 0.3	8.2 ± 0.02	0.46 ± 0.03	0.5 ± 0.01	0	0	0
Bergapten (50 mg/kg) + castor oil (10 ml/kg)	4.8 ± 0.3	6.7 ± 0.2	0.31 ± 0.05	0.42 ± 0.01	40.0***	67.39	81.7
Bergapten (100 mg/kg) + castor oil (10 ml/kg)	0 ± 0.0	1.6 ± 0.19	0 ± 0.0	0.25 ± 0.03	100***	0	19.51
Loperamide (2 mg/kg) + castor oil (10 ml/kg)	0 ± 0.0	2.2 ± 0.2	0 ± 0.0	0.11 ± 0.03	100***	0	22

Values are expressed as mean ± SEM *n* = 5. One-way analysis of variance (ANOVA) followed by *post hoc* Tukey’s test, ***p < 0.001 vs. castor oil group.

### 3.5 Effect of bergapten on intestinal fluid accumulation

**FIGURE 3:**
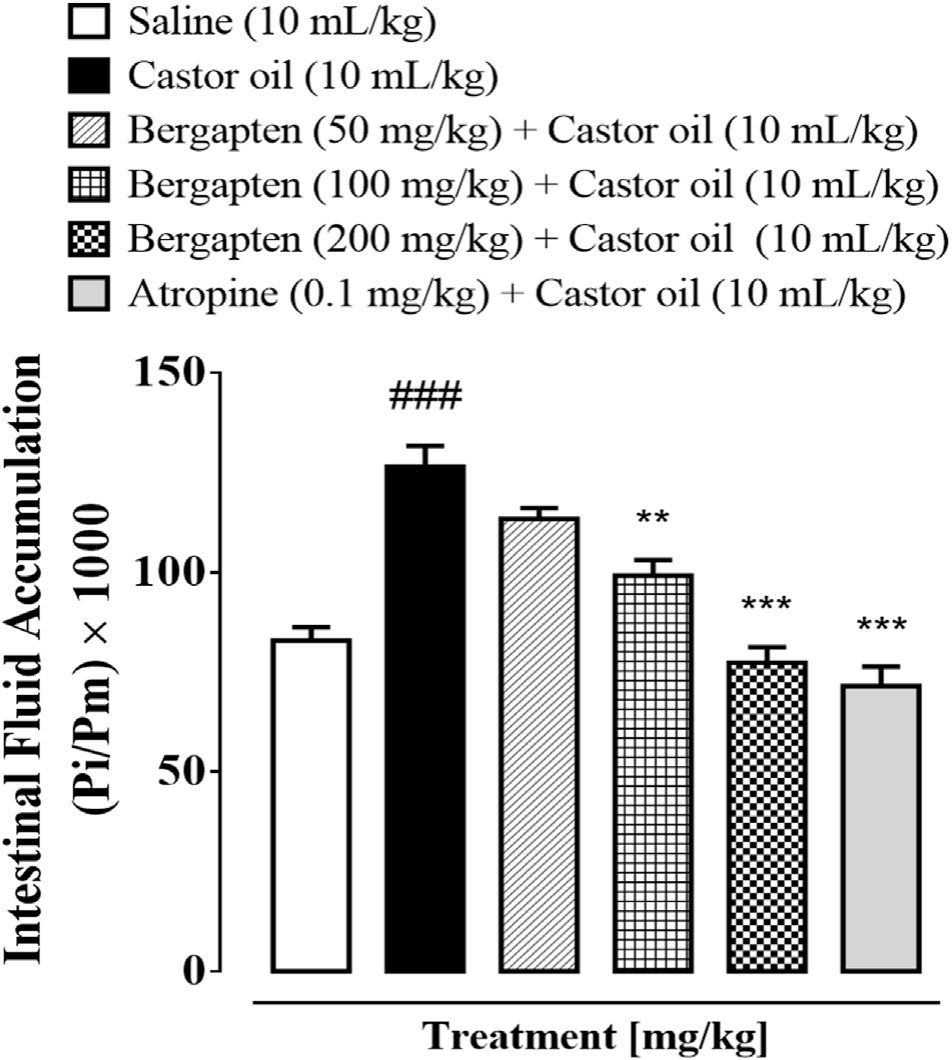
Inhibitory effect of bergapten and atropine on castor oil–induced fluid accumulation in mice. The anti-secretory effect is expressed as Pi/Pm x 1000 (g), where Pi is the weight of the small intestine and Pm is the weight of mouse. Results are expressed as mean ± SEM, *n* = 5. One-way analysis of variance with *post hoc* Tukey’s test. ^###^ *p* < 0.001 vs. saline group, **p < 0.01 vs. castor oil group, ***p < 0.001 vs. castor oil group.

When mice were given castor oil, which caused their intestines to fill up with fluid, 50–200 mg/kg of bergapten stopped the fluid from leaking out. Intestinal fluid buildup in the saline group was 86.5 1.2 (mean SEM, *n* = 5), but in the castor oil group, it was 124.16 1.96 (^###^ *p* < 0.001 vs. saline group). The amount of fluid buildup caused by castor oil was cut by bergapten at doses of 50, 100, and 200 mg/kg (**p < 0.01 vs. castor oil group), 95.84 1.5 (***p < 0.001 vs. castor oil group), and 75.98 0.91 (***p < 0.001 vs. castor oil group). Figure 3 shows that 10 mg/kg of atropine decreased the amount of fluid in the gut to 72.71 1.2 (***p < 0.001 vs. the castor oil group).

### 3.6 Effect on charcoal meal transit time

A dose-dependent delay in the passage of the charcoal meal through the small intestine is caused by bergapten. The saline group covered a distance of 94 cm. Bergapten at doses of 50, 100, and 200 mg/kg inhibited the transit of charcoal meal by 31.26%, 38.92%, and 43.01%, respectively (***p < 0.001 vs. charcoal group). The inhibitory effect of atropine (0.1 mg/kg) is 81.40% (***p < 0.001 vs. charcoal group) Table 2.

**TABLE 2:** Effect of bergapten and atropine on charcoal meal transit time in rats.

Treatment	Mean length of intestine (cm)	Distance moved by charcoal (cm)	Peristaltic index (PI) %	% inhibition
Saline (10 ml/kg)	94	0	0	0
charcoal (25 mg/kg)	92.6	90	96.9^###^	0
Bergapten (50 mg/kg) + charcoal (25 mg/kg)	81.2	54.2	66.74	31.26*
Bergapten (100 mg/kg) + charcoal (25 mg/kg)	81.2	50.8	59.3	38.92***
Bergapten (200 mg/kg) + charcoal (25 mg/kg)	91.8	48.2	55.33	43.01***
Atropine (0.1 mg/kg, i.p.) + charcoal (25 mg/kg)	90.8	16.4	18.06	81.40***

Values are expressed as mean ± SEM (*n* = 5). One-way analysis of variance (ANOVA) followed by *post hoc* Tukey’s test.^###^ *p <* 0.001 vs. saline group, *p < 0.05, ***p < 0.001 vs. charcoal group.

### 3.7 Effect of bergapten on spontaneous, K+-induced contractions and CCB effect

**FIGURE 4:**
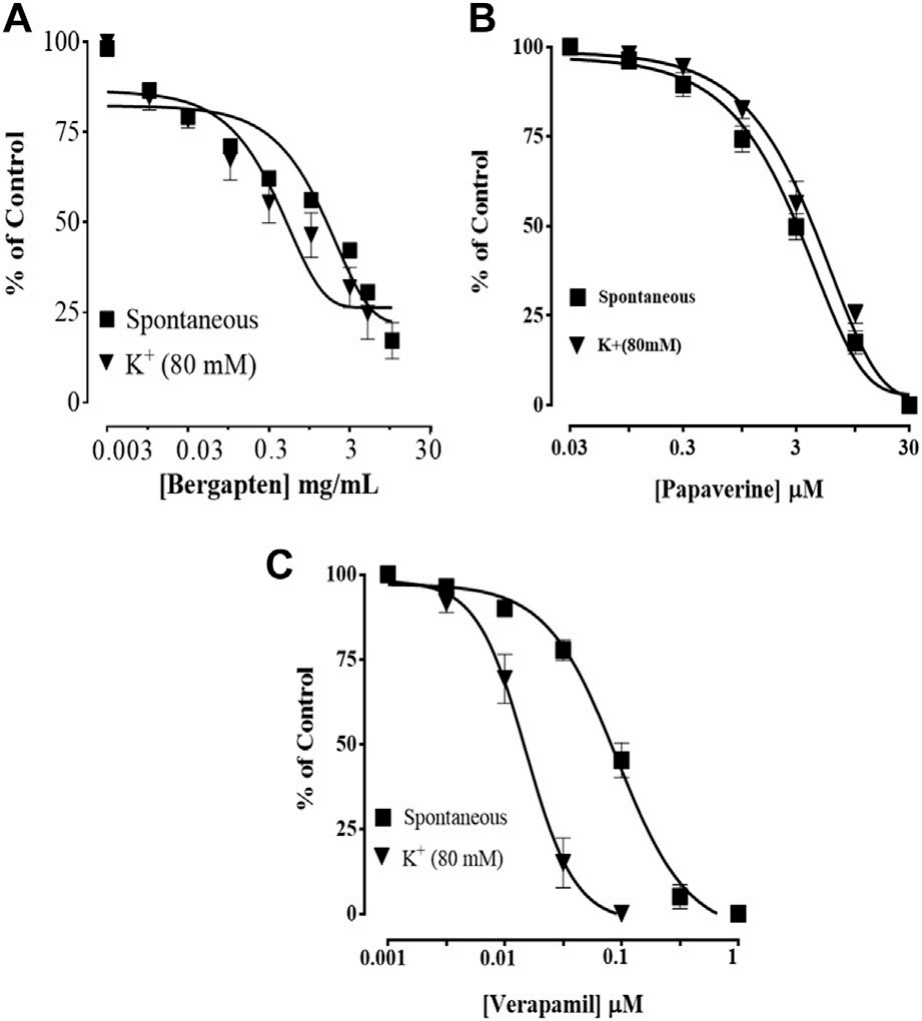
Concentration-dependent inhibitory effect on spontaneous and K^+^ (80 mM)-induced contractions of **(A)** bergapten, **(B)** papaverine, and **(C)** verapamil in isolated tissue preparations. The result expressed as mean ± SEM, *n* = 3–5.

**FIGURE 5:**
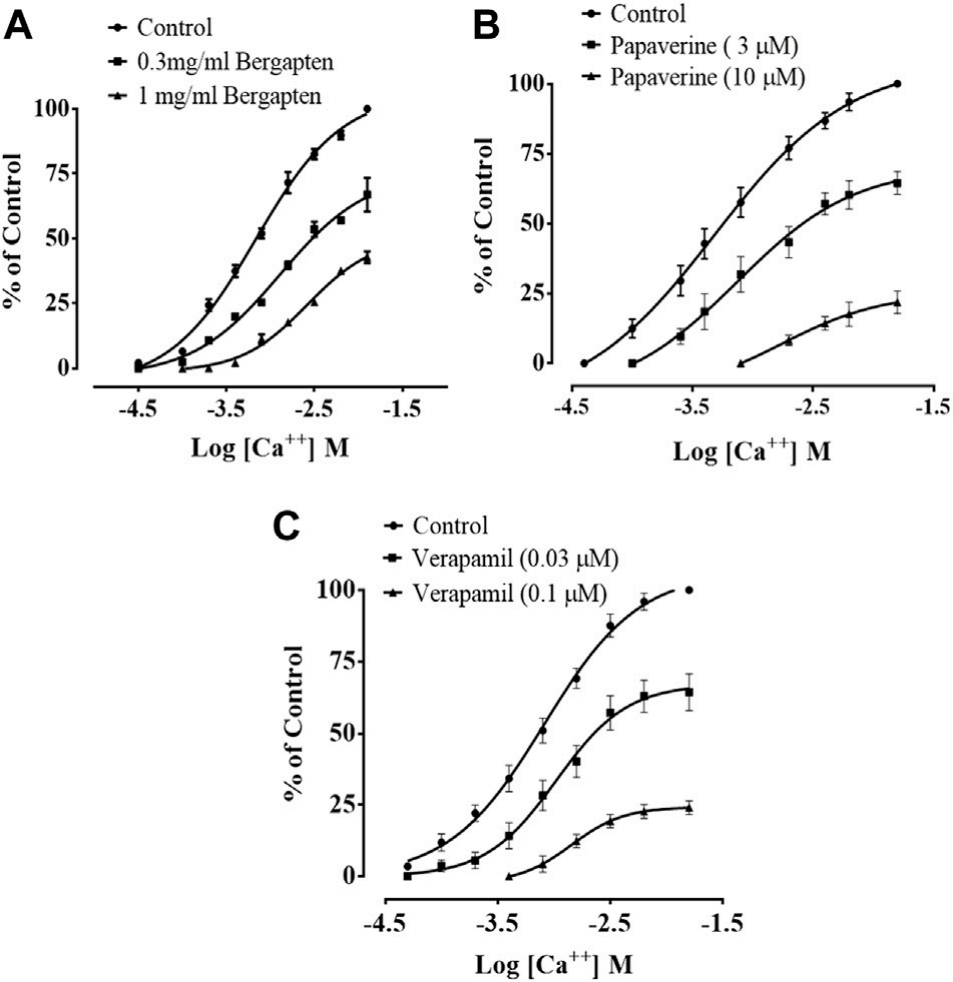
Effect of bergapten on concentration–response curves of Ca^2+^ in the absence and presence of increasing concentrations of **(A)** bergapten, **(B)** papaverine, and **(C)** verapamil in isolated rabbit jejunum preparations. The values shown are mean ± SEM, *n* = 3–4.

Figure 4 shows how bergapten, papaverine, and verapamil stop spontaneous and K^+^ (80 mM)-induced contractions, with EC50 values of 0.0026 mg/ml (1.69–2.82, *n* = 4) and 0.0139 mg/ml (1.68–18.9, *n* = 4), respectively. Figure 4A shows that both natural contractions and contractions caused by K^+^ (80 mM) were stopped by bergapten. Papaverine displayed non-specific inhibition in Figure 4B, with EC50 values of 0.4 μM (0.2–0.8, *n* = 4) for spontaneous contractions and 0.6 (0.3–1.3, *n* = 4) for high K^+^ trigger contractions. Verapamil had an EC50 of 0.04 M (0.03–0.06, *n* = 4) against spontaneous contractions and 0.12 M (0.10–0.20, *n* = 3) against K^+^-induced contractions (Figure 4C). Figure 5 demonstrates that when it was evaluated for calcium channel interaction, it shifted Ca^2+^ CRCs to the right, such as in papaverine and verapamil.

### 3.8 Effect on *H. pylori*

The zone of inhibition and MIC values were assessed against *H. pylori* isolate, which showed potent sensitivity by disc diffusion method for bergapten. It showed zone of inhibition of 30 ± 0.22 mm against isolate. The MIC value of 1.25 mg/ml was obtained (Supplementary Figure S16).

### 3.9 Effect on ethanol-induced ulcer

**FIGURE 6:**
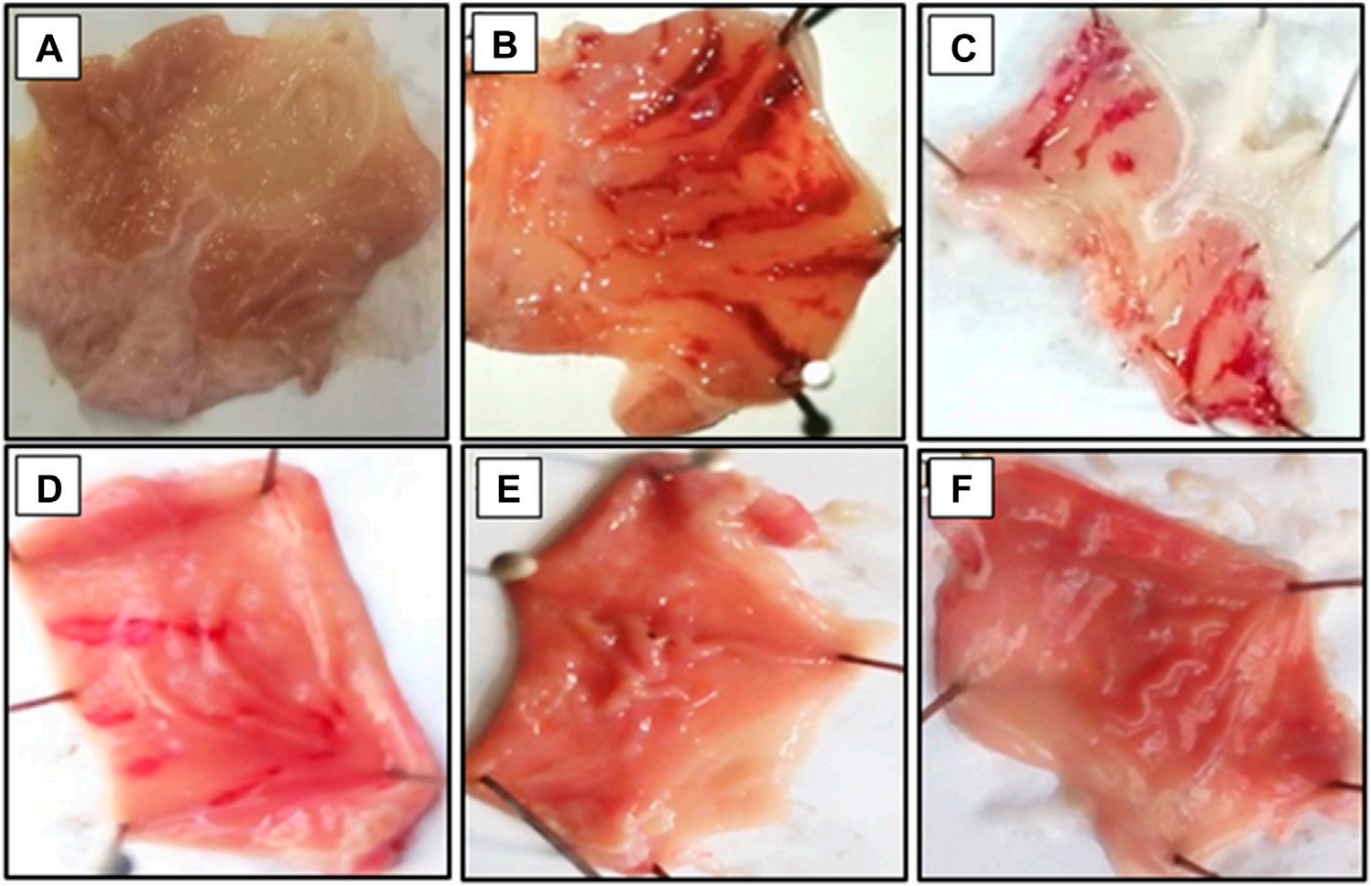
Gross-appearance of gastric mucosa in rat: **(A)** pre-treated with saline, 10 mL/kg, **(B)** Severe injuries are seen, as ethanol (1 mL/100 g) produced excessive hemorrhagic necrosis of gastric-mucosa **(C–E)** pretreated with bergapten at doses of 100, 150, and 200 mg/kg and **(F)** pretreated with omeprazole (20 mg/kg). The injuries reduce with increase of doses and omeprazole compare with ethanol treated group. At 200 mg/kg, bergapten showed most efficacious gastro- protective action.

Bergapten, at doses ranging from 100 to 200 mg/kg, showed anti-ulcer activity in a dose-dependent manner. At dosages of 50, 100, and 200 mg/kg, bergapten induced inhibition that was, respectively, 20 percent, 56 percent, and 74 percent (**p < 0.01, ***p < 0.001 vs. the saline group). An inhibitory impact of 86 percent was achieved with 20 mg/kg of omeprazole (Table 3). The macroscopical examination revealed the presence of the rats’ stomach mucosa (Figure 6).

**TABLE 3:** Protective effect of bergapten and omeprazole against ethanol-induced gastric ulcers in rats.

Treatment	Ulcer index	% inhibition
Saline 10 ml/kg	0 ± 0.0	−
Ethanol (1 ml/100 g)	10.0 ± 0.3^###^	0
Bergapten (50 mg/kg) + ethanol (1 ml/100 g)	8.0 ± 0.13**	20
Bergapten (100 mg/kg) + ethanol (1 ml/100 g)	4.4 ± 0.21***	56
Bergapten (200 mg/kg) + ethanol (1 ml/100 g)	2.6 ± 0.3***	74
Omeprazole (20 mg/kg) + ethanol (1 ml/100 g)	1.4 ± 0.14***	86

Values are expressed as mean ± SEM *n* = 5. One-way analysis of variance ANOVA, followed by *post hoc* Tukey’s test^###^ *p <* 0.001 vs. saline group, **p < 0.01, ***p < 0.001 vs. ethanol group.

### 3.10 Effect on H^+^/K^+^ ATPase activity

**FIGURE 7:**
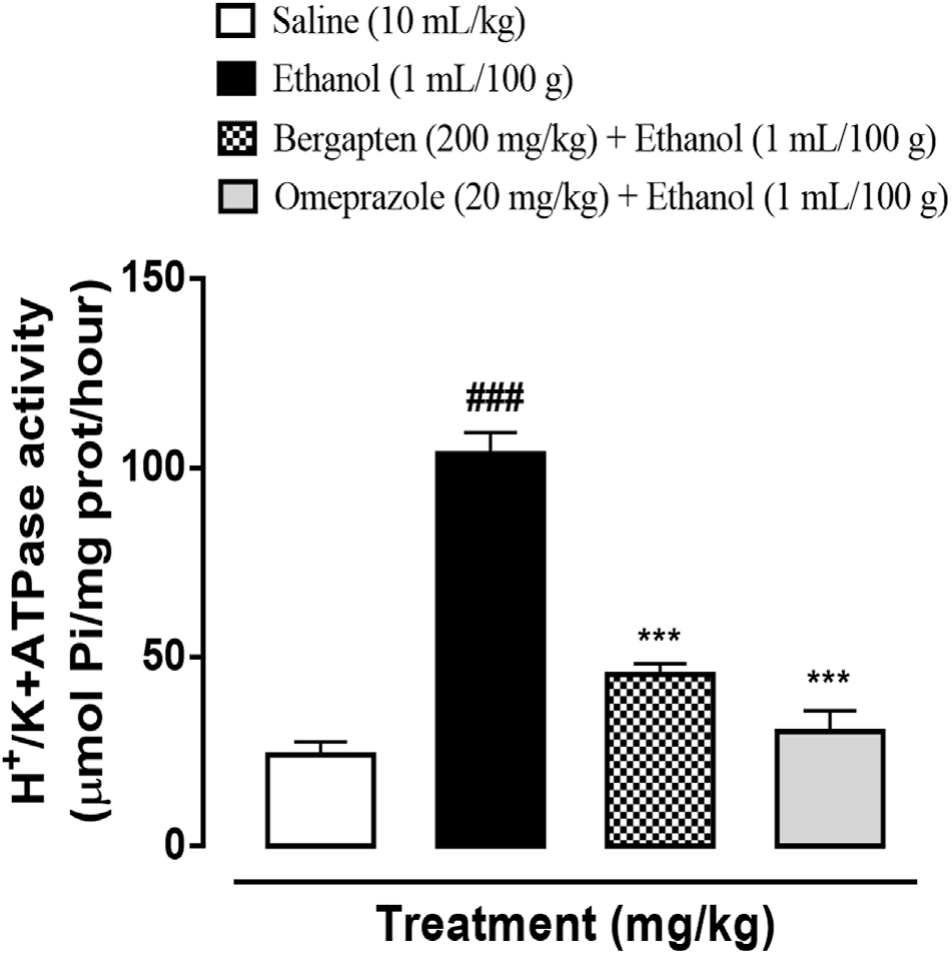
Effect of bergapten and omeprazole on decreasing rat gastric H^+^/K^+^ ATPase activity in the ethanol-induced gastric ulcer model. The data values are expressed as mean ± SEM (*n* = 3). One-way analysis of variance with *post hoc* Tukey’s test. ^###^ *p* < 0.001 vs. saline group, ***p < 0.001 vs. ethanol group.

The ethanol-induced group demonstrated significant increase in H^+^/K^+^ ATPase activity proving the release of acids in the stomach (^*###*^ *p* < 0*.*001 *versus* saline group). The treatment groups including bergapten (200 mg/kg) and omeprazole (20 mg/kg) significantly reduced the pump activity (***p < 0*.*001 *versus* ethanol group) shown in Figure 7.

### 3.11 Effect on oxidative stress markers

**FIGURE 8:**
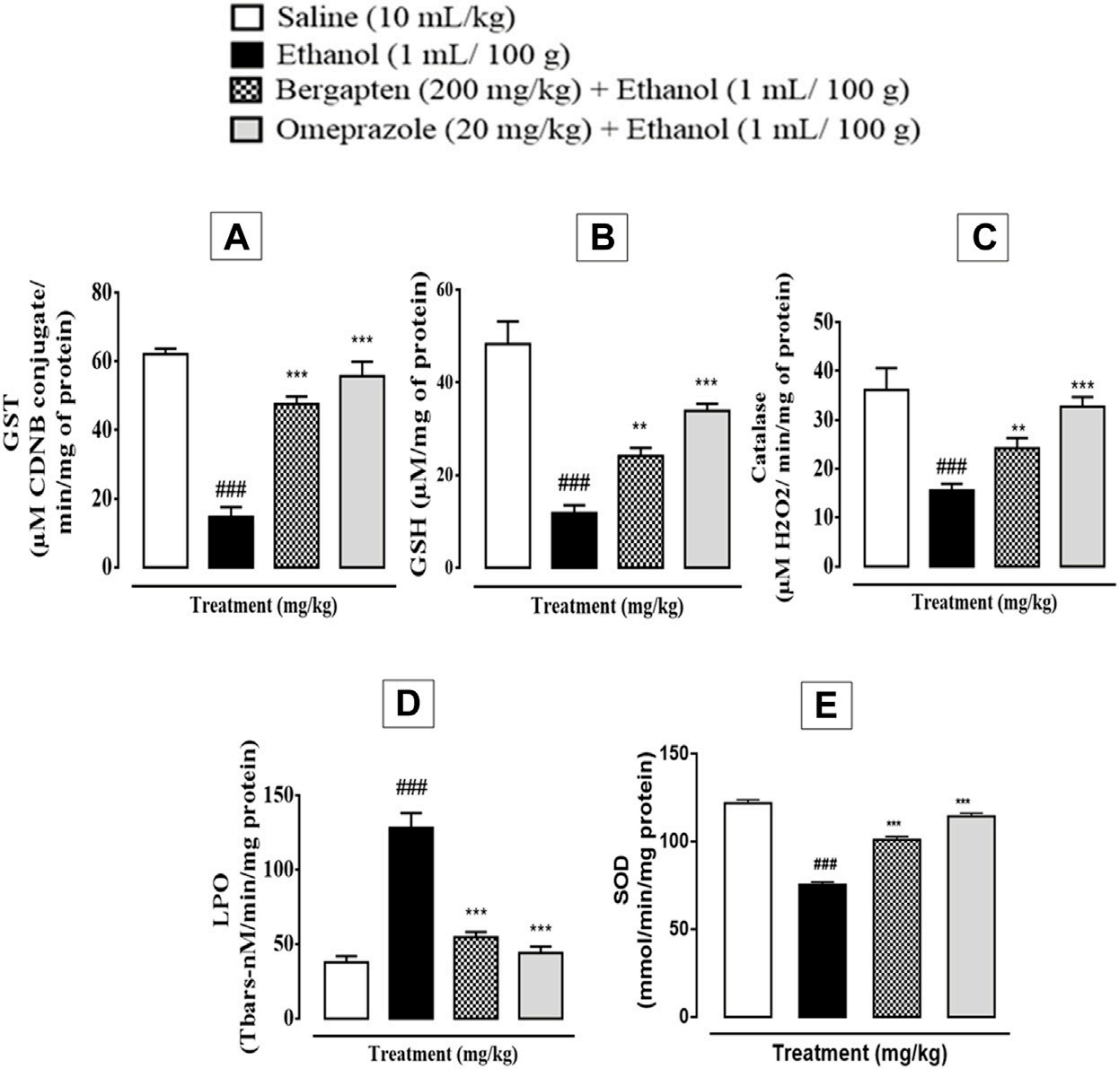
Effect of bergapten (200 mg/kg) and omeprazole groups against **(A)** glutathione-S-transferase (GST), **(B)** glutathione (GSH), **(C)** catalase, **(D)** lipid peroxidation (LPO), and **(E)** superoxide dismutase (SOD) in the ethanol-induced gastric ulcer model. The data are expressed as mean ± SEM (*n* = 3), and analyzed by one-way ANOVA with *post hoc* Tukey’s test. ^###^ *p* < 0.001 vs. saline group, ***p < 0.001, **p < 0.01 vs. ethanol group.

In ethanol-induced ulcer stomach tissues, the activity of GST, GSH, and catalase was dramatically decreased, while LPO was elevated (^###^ *p* < 0.001 in comparison to the saline group), which is a clear indication of oxidative stress. However, the levels of GST (***p < 0.001 in comparison to the ethanol group), GSH (**p < 0.01, ***p < 0.001 in comparison to the ethanol group), and catalase (***p < 0.001 in comparison to the ethanol group) were significantly increased in the treatment groups that included bergapten (200 mg/kg) and omeprazole (20 mg/kg). Figure 8 shows that there was a substantial reduction in LPO levels across the treatment groups (***p < 0.001 in comparison to the ethanol group). Superoxide dismutase activity indicated significant increase in SOD activity in the bergapten-treated group (***p < 0.001 in comparison to the ethanol group) as compared to reduced activity in the ethanol group. This effect was similar to that produced by standard drug omeprazole (***p < 0.001 in comparison to the ethanol group).

### 3.12 Histopathological analysis

**FIGURE 9:**
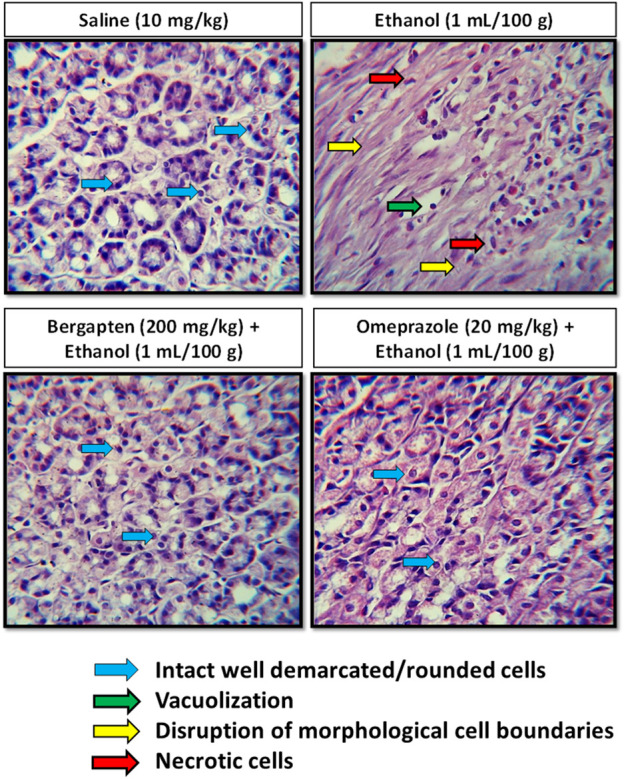
Histopathological slides of gastric tissues showing effect of pretreatment with **(A)** saline (10 ml/kg), **(B)** ethanol (1 ml/100 g), **(C)** bergapten (200 mg/kg), and **(D)** omeprazole (20 mg/kg) bergapten and omeprazole in the ethanol-induced ulcer model.

Hematoxylin and eosin (H&E) staining was performed in order to distinguish among necrotic cells from intact one after administration of ethanol in stomach for further examination of gastroprotective effect of bergapten, as shown in Figure 9. In the stomach’s most vulnerable regions, ethanol causes significant cellular alterations, whereas bergapten treatment lessens the damage. In comparison to the ethanol-treated group, a significant difference was seen in the control group. The injured region exhibits changed mucosal cell size and shape and aberrant color staining. Control animals’ histology preparations indicated no alterations. Bergapten-treated groups had more viable cells than ethanol-treated ones.

### 3.13 Effect on TNF-α, PGE_2_, and IL-8 expression

**FIGURE 10:**
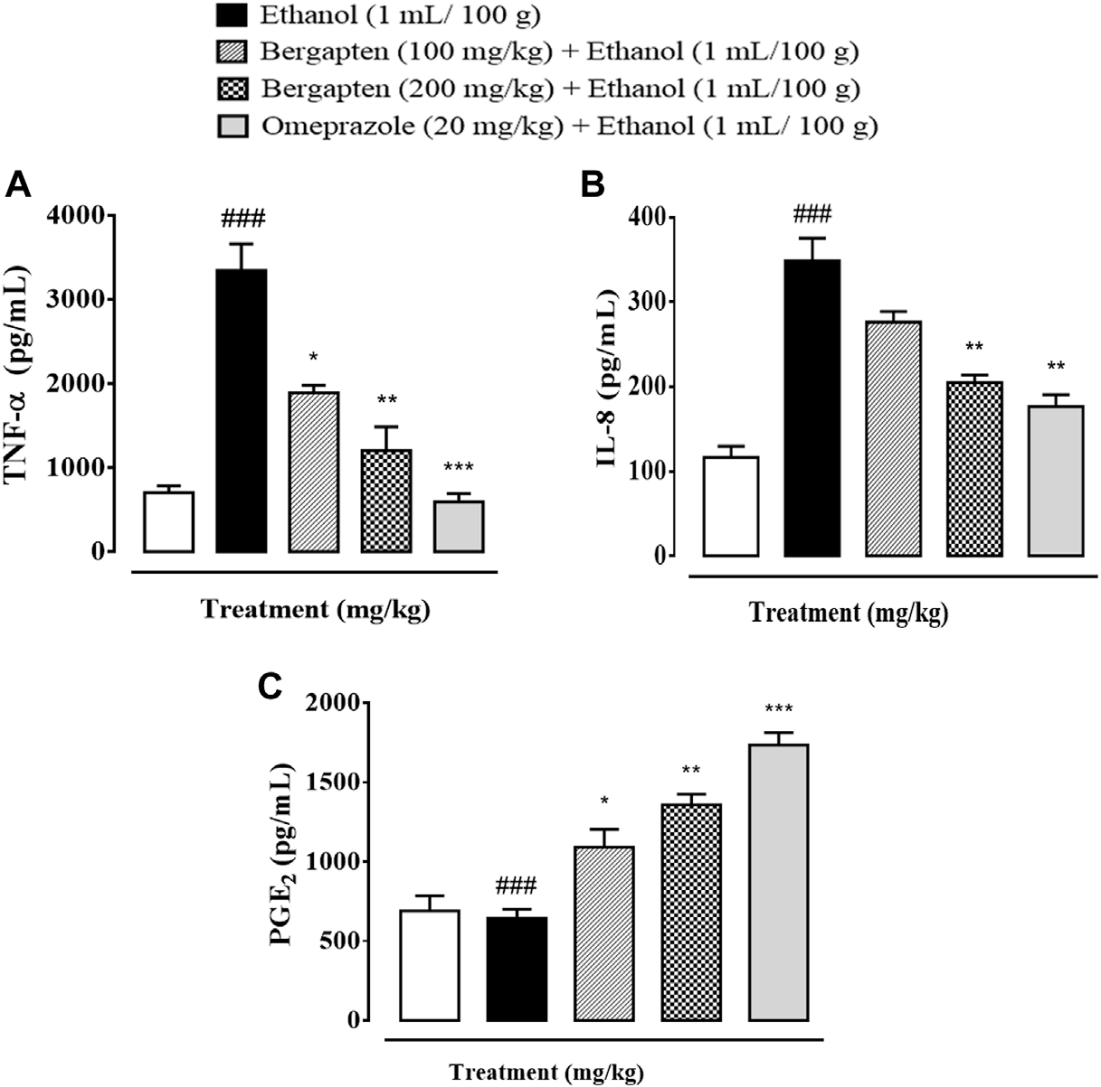
Effect of bergapten and omeprazole on **(A)** TNF-α (tumor necrosis factor alpha), **(B)** IL-8 (interleukin-8), and **(C)** PGE_2_ (prostaglandin-E_2_) expression in wound healing in the ethanol-induced gastric ulcer model examined by ELISA. The data are expressed as mean ± SEM (*n* = 3). One-way ANOVA with *post hoc* Tukey’s test. ^###^ *p* < 0.001 vs. saline group, ***p < 0.001, **p < 0.01, *p < 0.05 vs. ethanol group.

Figure 10 depicts the effect of bergapten on TNF-α, PGE2, and IL-8 in the stomach of an ethanol-induced ulcer model. Bergapten substantially reduced the expression of TNF-α, PGE_2_, and IL-8 in both treatment groups as compared to the ethanol control group, according to a one-way ANOVA and *post hoc* Tukey’s test. The results show a considerable difference between the treated group and the disease group. The degree of inflammatory mediator release from tissue in bergapten at doses of 100 and 200 mg/kg was considerably lower at higher doses. In contrast, bergapten has more pronounced effect with respect to omeprazole.

### 3.14 Western blot analysis

**FIGURE 11:**
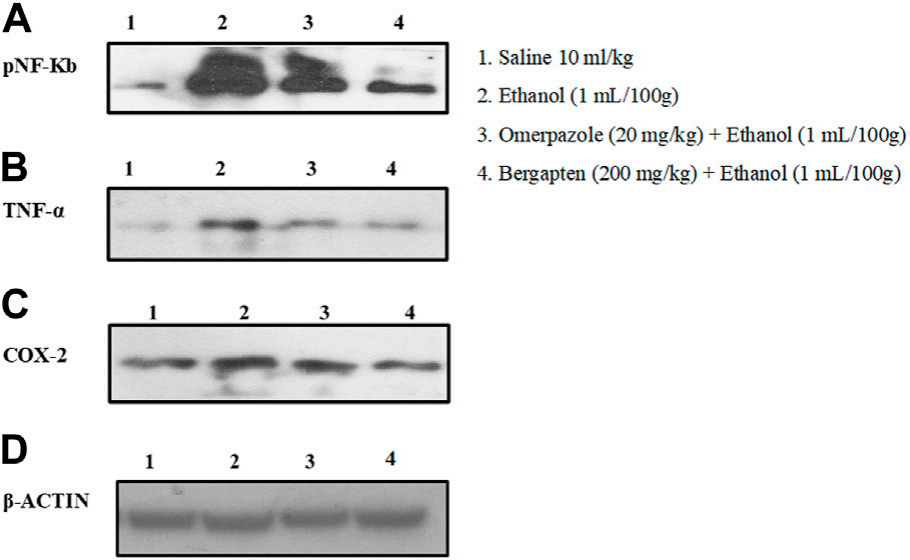
Pictorial representation showing inhibitory effect of bergapten and omeprazole groups against **(A)** phosphorylated nuclear factor kappa B (pNF-Kb), **(B)** tumor necrosis factor alpha (TNF-α), **(C)** cyclooxygenase 2 (COX-2), and **(D)** β-actin expression in ethanol-treated gastric tissues using western blot techniques, where 1 indicates saline group, 2 indicates the expression in ethanol groups, 3 indicates omeprazole group, and 5 indicates bergapten (200 mg/kg) group.

**FIGURE 12:**
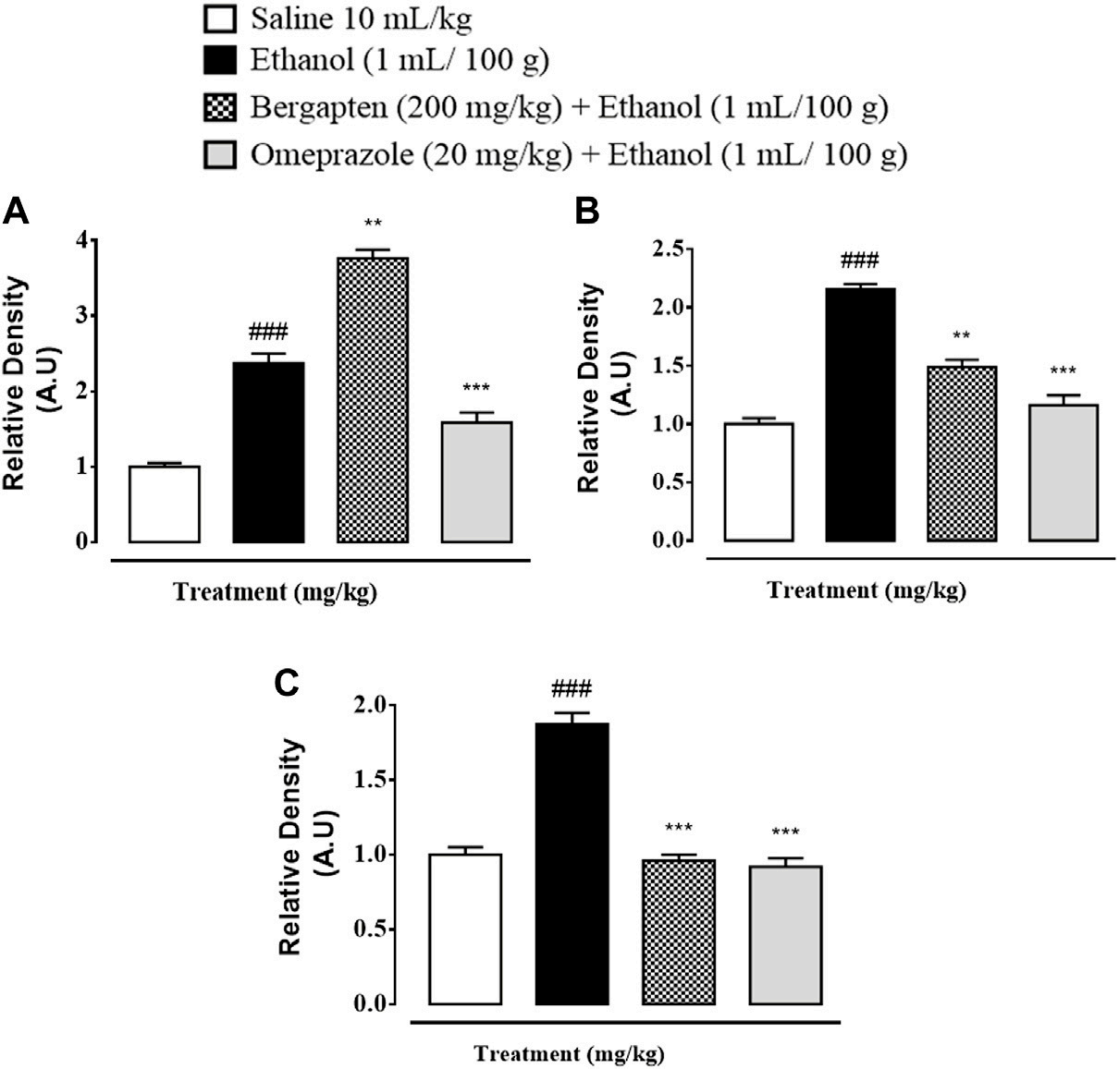
Graphical representation showing inhibitory effect of bergapten and omeprazole groups against **(A)** phosphorylated nuclear factor kappa B (pNF-Kb), **(B)** tumor necrosis factor alpha (TNF-α), and **(C)** cyclooxyegenase 2 (COX-2) in ethanol-treated gastric tissues using western blot techniques. The data are expressed as mean ± SEM (*n* = 3). One-way ANOVA with *post hoc* Tukey’s test. ^###^ *p* < 0.001 vs. saline group, ***p < 0.001, **p < 0.01 vs. ethanol group.

Western blot analysis (gastric tissues) confirmed that COX-2, TNF, and p-NFƙB levels were increased in the ethanol-treated group (^###^ *p* < 0.001 versus saline group), but bergapten (200 mg/kg)- and omeprazole (20 mg/kg)-treated groups showed decreased expression levels as compared to the negative control group (***p < 0.001 vs. ethanol group) Figures 11, 12.

### 3.15 Quantification of mRNA levels

**FIGURE 13:**
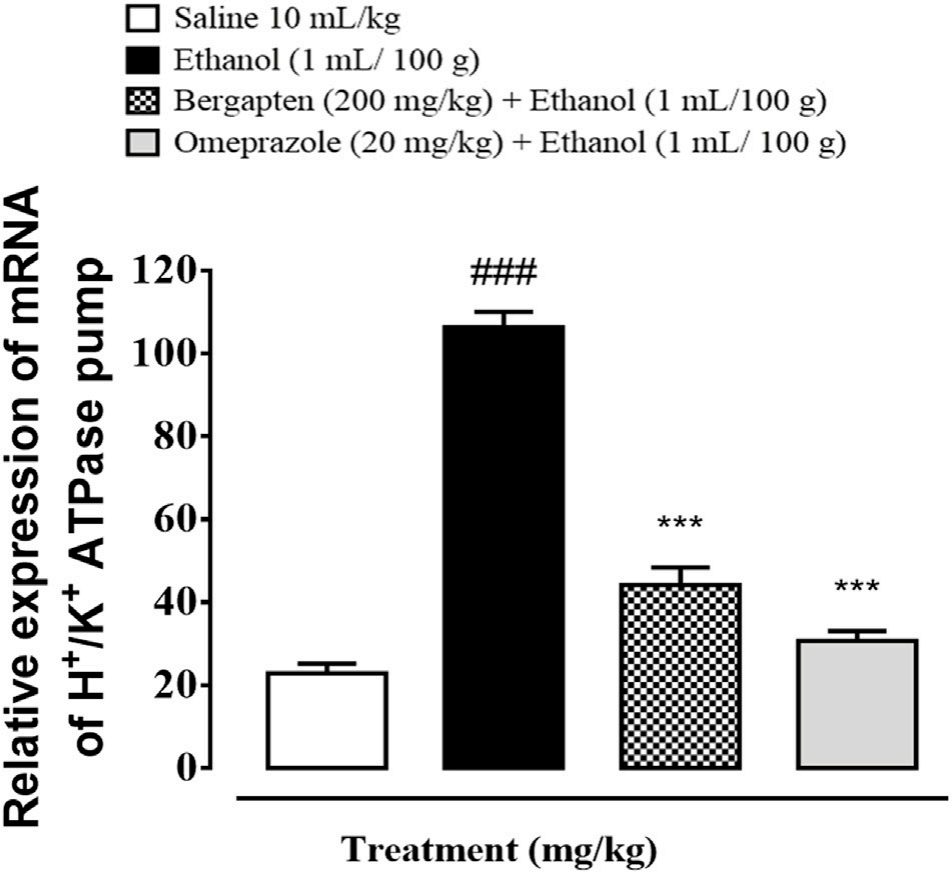
Graphical representation indicating effect of bergapten and omeprazole groups against mRNA expression of H^+^/K^+^ ATPase in ethanol-treated gastric tissues, using reverse transcriptase polymerase chain reaction (RT-PCR) technique. The data are expressed as mean ± SEM (*n* = 3). One-way ANOVA with *post hoc* Tukey’s test. ^###^ *p* < 0.001 vs. saline group, ***p < 0.001 vs. ethanol group.

RT-PCR determined the fold expression of H^+^/K^+^ ATPase in the ethanol-induced gastric ulcer model (Figure 13). The ethanol (1 ml/100)-administered group indicate increased expression of H^+^/K^+^ ATPase mRNA levels (^###^ *p <* 0.001 *versus* saline group). Bergapten (200 mg/kg) caused a significant decrease in expression levels (***p < 0*.*001 vs. ethanol group). Omeprazole (20 mg/kg) also reduced the expression comparative to negative control group (***p < 0*.*001 vs. ethanol group).

### 3.16 Toxicity studies

The OECD guidelines 425 were used to evaluate the safety profile of bergapten. After ingesting 2000 mg/kg of compound, the animal’s fur and skin, feces consistency, urine’s color, its breathing rhythm, and its sleeping patterns were all normal. There were no convulsions or signs of discomfort in any of the animals. Both groups’ weights increased properly over the course of the 14-day treatment. The effect on behavioral parameters is given in Table 4, and the effect of 2000 mg/kg dose of bergapten on organ weight is given in Table 5. When comparing the weight of organs in drug-treated animals to the control group, no significant differences were identified.

**TABLE 4:** Behavioral patterns of rats in bergapten-treated (2000 mg/kg p.o.) and control groups.

Parameters	30 min		4 h		24 h		48 h		7 days		14 days	
	CG	TG	CG	TG	CG	TG	CG	TG	CG	TG	CG	TG
Fur and skin	N	N	N	N	N	N	N	N	N	N	N	N
Itching	−	+	−	+	−	−	−	−	−	−	−	−
Eyes	N	N	N	N	N	N	N	N	N	N	N	N
Salivation	N	N	N	N	N	N	N	N	N	N	N	N
Mucous membrane	N	N	N	N	N	N	N	N	N	N	N	N
Respiration	N	↑	N	↑	N	N	N	N	N	N	N	N
Urination (color)	N	N	N	N	N	N	N	N	N	N	N	N
Faeces consistency	N	N	N	N	N	N	N	N	N	N	N	N
Convulsions and tremers	−	+	−	+	−	−	−	−	−	−	−	−
Sleep	N	N	N	N	N	N	N	N	N	N	N	N
Coma	−	−	−	−	−	−	−	−	−	−	−	−
Mortality	−	−	−	−	−	−	−	−	−	−	−	−

CG, control group; TG, bergapten-treated groups, N, normal; +, present; ↑, Increased; −, not found.

**TABLE 5:** Effect of bergapten on the organ weight of treated and control rats (at limit dose 2000 mg/kg b.w. p.o).

Organs	Control	Bergapten (2000 mg/kg)
Heart	0.33 ± 0.3 g	0.38 ± 0.4 g
Kidney	1.0 ± 0.2 g	1.3 ± 0.2 g
Liver	6.4 ± 0.3 g	6.5 ± 0.5 g
Brain	1.39 ± 0.2 g	1.41 ± 0.1 g

#### 3.16.1 Biochemical analysis

**FIGURE 14:**
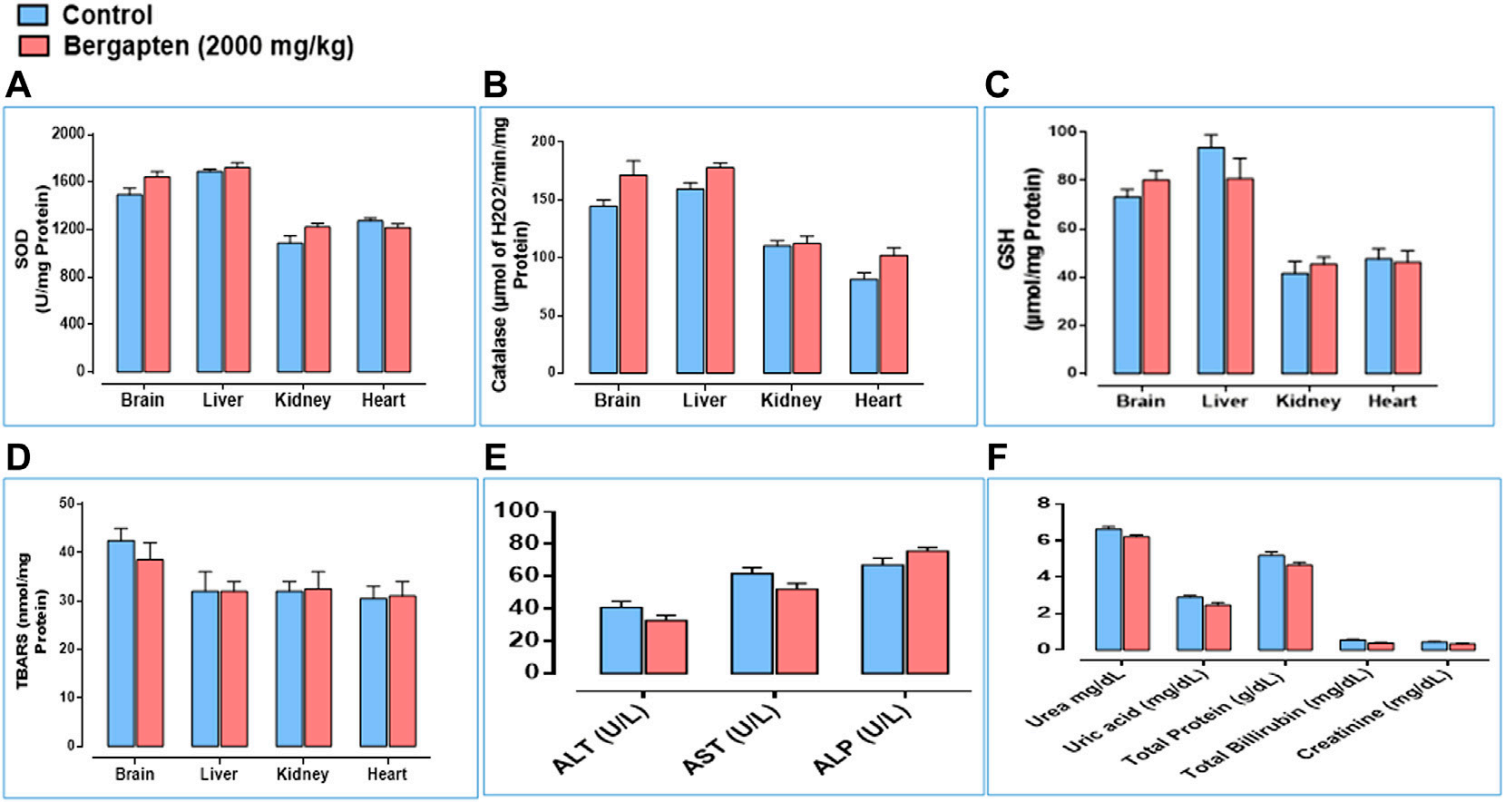
Toxicology assay findings showing **(A)** the expression level of SOD (superoxide dismutase), **(B)** expression level of catalase, **(C)** expression level of GSH (glutathione), **(D)** expression of TBARS (thiobarbituric acid reactive substances), **(E)** effect on liver function tests such as ALT (alanine transaminase), AST (aspartate aminotransferase), and ALP (alanine phosphatase). **(F)** Renal function test (RFTs) measuring urea, uric acid, total protein, total bilirubin, and creatinine levels in control and bergapten (2000 mg/kg)-treated rats.

Effect on LFTs, RFTs, and oxidative stress markers are given in Figure 14. ALT and AST levels decreased in the bergapten group and ALP levels increased compared to the control group. RFTs indicated no renal toxicity signs based on RFTs outcome. Oxidative stress markers such as SOD, catalase, and GSH levels increased in the brain, kidney, and heart; however, GSH decreased in the liver indicating mild liver toxicity.

#### 3.16.2 Hematological analysis

Effect on hematological parameters are given in Table 6. Bergapten showed a stable hematology profile. There was no evidence of hematological toxicity.

**TABLE 6:** Effect of bergapten (at limit dose 2000 mg/kg b.w. p.o) on the complete blood count (CBC) and lipid profile of control group rats.

Parameters	Unit	Control	Bergapten (2000 mg/kg)
CBC			
Hb	g/dL	10.5 ± 0.1	10.9 ± 0.2
Total RBC	*10^6^/uL	4.95 ± 0.4	5.2 ± 0.05
WBC count (TLC)	*10^3^/uL	2.2 ± 0.11	3.5 ± 0.2
Platelets	*10^5^/uL	256 ± 0.5	221 ± 1.9
HCT	%	31.3 ± 0.6	36.6 ± 0.2
MCV	Fl	50.9 ± 0.05	47.1 ± 0.9
MCHC	g/dL	34 ± 0.22	27.3 ± 0.6
Neutrophils	%	9.9 ± 0.8	10.2 ± 0.5
Lymphocytes	%	88 ± 1.4	95 ± 1.5
Monocytes	%	4 ± 0.12	3 ± 0.08
Eosinophils	%	2 ± 0.11	1 ± 0.04
MCH	Pg	15.7 ± 0.6	16 ± 0.018
Lipid profile			
Cholesterol	mg/dl	155 ± 1.5	170 ± 1.8
Triglycerides	mg/dl	130 ± 1	145 ± 1.2
H.D.L (cholesterol)	mg/dl	27 ± 0.68	31 ± 0.53
L.D.L (cholesterol)	mg/dl	107 ± 1.24	115 ± 0.78
V.L.D.L	mg/dl	30 ± 0.45	25 ± 1.2

#### 3.16.3 Histopathological analysis

**FIGURE 15:**
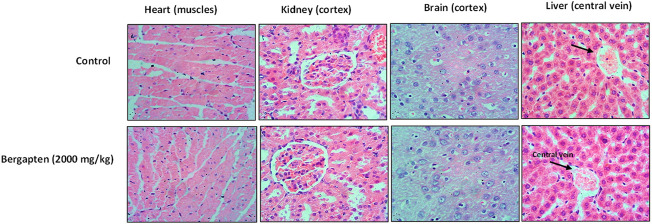
Hitopathological slides showing the effect of bergapten on vital organs of rats using hematoxylin and eosin staining histopathological method. Bar 50 µm, magnification × 40.

There was no evidence of vacoulation, dystrophy, or atrophy in key organs such as the brain, liver, kidney, and heart according to histopathological investigation given in Figure 15.

## 4 Discussion

Based on a review of the literature on use of bergapten and plants with bergapten as the main phytoconstituent in gastric disorders like gastritis, constipation, and diarrhea, different tests were used to evaluate bergapten’s anti-ulcer, anti-secretory, charcoal meal transit time, gastrointestinal motility, and anti-ulcer effects. An *in vitro*, *in silico*, *in vivo*, and proteomic approach was used for the explication of possible underlying mechanism(s) to rationalize aforementioned GIT ailments. Docking is a preliminary way of determining a ligand’s affinity for a specific protein target (Zhang et al., 2021). Drug research and development heavily rely on molecular docking, which also determines target specificity and performs structure-based evaluation (Zhou et al., 2020; Faheem et al., 2021). For *in silico* studies initially bergapten was docked against targets involved in GI ailments. Based on positive results, molecular docking simulation studies were conducted on the targets chosen for molecular studies, which included voltage-gated calcium channels (PDB ID: 1t3s) and H^+^/K^+^ ATPase (PDB ID:4ux2). This helped to strengthen the *in silico* results. Researchers have used PDB IDs from the Research Collaborator for Structural Bioinformatics Protein Database (RCSB PDB) to study the aforementioned target proteins in GI diseases (Pecsi et al., 2010). Docking analysis and molecular dynamic simulation studies were used to figure out the best positions. MD simulations let you see how molecules change shape, where their atoms are, and how they interact with their surroundings. The changes in these shapes helps access the structural interactions resulting best pose for ligand and protein complexes. This information is helpful to identify active pockets and poses that can be used to design ligands to best fit the target protein (Karplus and McCammon, 2002; Hernandez et al., 2016). Simulations were run for 100 ns in this study. The interactions were found to be stable, and the RMSF and RMSD values showed that targets proteins and bergapten worked well together. Less changes in RMSD show that interactions are strong (Faheem et al., 2021). This lead to the basis for further taking this research to animal testing by applying *in vivo* models.

To assess the effect of bergapten in gastrointestinal disorders such as castor oil-induced diarrhea, this study was design to measure the antidiarrheal effect of bergapten in rats similar to effect produced by loperamide, a standard drug while its possibly underlying mechanism were estimated through isolated tissue preparations also associated with reduction in gastric motility (Qazi et al., 2022). Castor oil is responsible for increasing intestinal fluid as well as diarrheal effect through its active metabolite, that is, ricinoleic acid. It changes the electrolyte and water transport and generates enormous contractions in the transverse and distal colon (Ansari et al., 2019). Bergapten acted as gut relaxant, observed antidiarrheal effects in the animal model. The calculated antidiarrheal effect of bergapten in rats, mediated possibly through the blockade of Ca^2+^ channels, evidence for its effectiveness in diarrhea, though additional mechanism(s) cannot be ruled out (Rehman et al., 2012). For charcoal meal transient time activity, bergapten in the small intestinal produced suppression of propulsion of charcoal marker in test doses just like standard drug atropine sulfate which has anticholinergic effect on GI transit leading to reduced motility (Ansari et al., 2019). The reduced motility causes sufficient reabsorption of water and contents through the intestine which can also be correlated to antidiarrheal findings of bergapten in this study (Qazi et al., 2022). Other functional regulators involved in GI motility include acetylcholine, histamine, prostaglandins and ionic exchanges involving K^+^ and Ca^++^ ions (Sadraei et al., 2022). Usually spasmolytic agents relax the GI tone and therefore are also found beneficial therapeutically in diarrhea. *In vitro* analysis was performed to find out what might be going on with the GIT inhibitory mechanism and the gastric mucosa. Bergapten produces its relaxant effect through the inhibition of Ca^2+^ channels. To figure out how it works, calcium channel blocking activity was carried out. Therefore, the spasmolytic effect of bergapten was studied through isolated rabbit jejunum assay, where bergapten proved to suppress both spontaneous and K^+^ induced contractions. Bergapten pretreatment shifted the Ca^2+^ CRCs toward right similar to that of papaverine and verapamil that confirms the calcium channel blocking effect. Bergapten has the same Ca^2+^-blocking effect as verapamil, but at a slightly higher concentration. Bergapten has a relaxing effect on smooth muscles, which may be caused by a combination of anticholinergic and Ca^2+^ antagonist mechanisms, which provides sound mechanistic background for its application in future for the hyperactive gut and airways disorders, such as abdominal colic, diarrhea and asthma that have prominent anticholinergic effect on intestinal transit (Rehman et al., 2011; Wen et al., 2022)*.* A diminished gastric motility tone causes increase in stay of substances in intestine, which permits better water absorption*.* This finding proposes that bergapten has effect on the peristaltic movement of intestine which indicates its anti-motility activity results in anti-spasmodic properties*.* Similar effect to that of papaverine and verapamil standard calcium channel blocker. Ca^2+^ levels are increased in response to various stimuli released by the gut (such as histamine and prostaglandins), which stimulate the gut and eventually enhance its cytosolic concentration. In addition to non-targeted inhibitory action, the production of mediators that block the aforementioned channels is crucial (Mehmood et al., 2015).

Inflammation of the mucosal layer of the gastrointestinal (GI) tract is not only frequently associated with ulceration of those tissues, but it also plays a crucial role in both the formation and repair of the lesions (Raish et al., 2021). The mediators that orchestrate inflammatory responses can also affect the mucosa’s resistance to injury caused by noxious chemicals, while others make the mucosa more vulnerable to injury. In the past 3 decades, there has been a change in perspective with regard to GI mucosal defence, moving away from a focus on the toxic secretions inside the lumen of the GI tract and toward a focus on the tissue responses that compose what is referred to as “mucosal defence.” Nitric oxide, eicosanoids (prostaglandins, leukotrienes, and thromboxanes), neuropeptides, cytokines, and proteinases are among the essential GI mediators. These chemical mediators are possible targets for treatment (Mahmoud et al., 2019). In fact, targeting these mediators has recently shown to be successful in treating GI ulcerative/inflammatory disorders (Wallace and Chin, 1997). In gastrointestinal tract various aggressive and protective factors play important role in production and release of acid. Disturbance in these factors results in rupturing of mucosal protection which expose gastric lining to difference enzyme and acid production leading to the sores called ulcers (Tulassay and Herszényi, 2010). An ethanol-induced gastric model was used to explore the beneficial effect of bergapten through variety of mechanisms that stimulates ulcers including free radicals OH^−^, NO production, mucus exhaustion, mucosal damage, release of superoxide anion, which ultimately prolonged the tissue oxidative stress and release of inflammatory mediators like TNF-α, PGE_2_, and IL-8 (Wallace and Chin, 1997; Ansari et al., 2021). Bergapten showed marked inhibition on ethanol-induced gastric lesions in comparison to disease group. The potential of bergapten to produce anti-ulcer effect might be due to its CCB effect, as Ca^2+^ antagonist is well known to demonstrate such effects.

On the other hand, H^+^/K^+^ ATPase is an enzyme located in the stomach’s gastric membrane vesicles that catalyzes the exchange of intracellular H^+^ and external K^+^ as well as the hydrolysis of cytoplasmic ATP. Activation of the aforementioned pump causes release of protons causing acidic environment. This increase in acid production further damages the underlying mucosal membrane and its disruption may cause ulceration (Tulassay and Herszényi, 2010; He et al., 2018). Therefore, one of the therapeutic approaches is to reduce the acid production by inhibiting the H^+^/K^+^ ATPase pump in the gastric membrane. For evaluating effect of bergapten in this regard *in vitro* inhibitory assay was conducted which revealed that bergapten inhibited the H^+^/K^+^ ATPase pump effectively and comparable to the standard proton pump inhibitor available commercially, that is, Omeprazole. To further strengthen the results, RT-PCR study was conducted to reveal the expression levels of H^+^/K^+^ ATPase. It was confirmed further through RT-PCR that expression level of H^+^/K^+^ ATPase reduced significantly. By this we can confirm that underlying mechanism for gastroprotective anti-ulcer activity of bergapten is due to proton pump inhibition. In pathophysiology of gastric ulcers, oxidative stress plays a vital role. Antioxidant and nitric oxide free radical scavenging activity has been shown by bergapten, which may be responsible for its effectiveness as anti-ulcer agent and further validated by LPO, catalase, GST, GSH and SOD assay (Mehmood et al., 2015; Sisay et al., 2017; Liang et al., 2021). Increased oxidative stress provokes the release of pro-inflammatory mediators such as interleukin-8 (IL-8), TNF-α, COX-2 upregulation, and NF-ƙB activation that plays role in inflammatory cascade further aggaravating ulcer (Khan et al., 2011; Sisay et al., 2017). Furthermore, ELISA was done to quantify the protein expression of TNF-α, IL-8, and PGE-2. Bergapten has significantly attenuated necrosis in ulcerative tissue as compared to control achieved through proteomic analysis. Western blotting was also carried to evaluate the antiinflammtory response in ulcer. Bergapten protects the stomach of rats through inhibition of inflammatory markers such as TNF-α, p-NFκB, COX-2, and enhancing protective PGE2 response as illustrated in results of western blotting. The results were quite significant as compared to ethanol (*p* < 0.001 vs. normal saline). Histopathological examination also revealed the intact morphology of the bergapten pretreated groups indicating effectiveness in preventing ethanol-induced mucosal injury. In pathophysiology of gastric ulcers one of the major contributors include H. *pylori* infections and H^+^/K^+^ ATPase pump. H. *pylori* infection is treated using combination of antibiotics termed as triple and quad therapy, as use of a single antibiotic shows significant relapse of the infection (He et al., 2018). Therefore, there is need of an agent that may concomitantly scavenge the bacterial infection as well as reduces acid secretions. Disc diffusion method utilized indicated efficacy of bergapten in scavenging the bacterial growth and showing a prominent zone of inhibition. To access the safety profile of bergapten, toxicological investigation is carried out. Bergapten has been reported to cause photosensitivity and apart from this aspect further toxicology evaluation has not been reported yet. In current study, OECD 425 guidelines were followed for conducting animal studies for acute toxicity accessment of bergapten. In acute toxicity analysis oral dosing helps to access the clinical safety in terms of dose ranges (Saleem et al., 2017). Bergapten is however classified with precautionary codes and hazard markers by GHS (globally harmonized system of classification) for chemicals due to its irritant nature to skin and to inhalation hazard.

## 5 Conclusion

The relevance of bergapten to the pharmaceutical industry is shown by the fact that researchers are actively investigating its potential as a result of the antidiarrheal, anti-secretory, anti-spasmodic, anti-motility, and anti-ulcer qualities that it has been shown to possess. Bergapten showed a strong affinity for the H^+^/K^+^ ATPase pump and voltage-gated L-type calcium channels. Further molecular simulations confirmed the favorable interactions with the aforementioned targets making it a favorable ligand for gastroprotection. Bergapten also increases the protective GST, GSH, and catalase and reduces the expression of LPO. It shows gastroprotective capability by reducing the prevalence of ulcer after ethanol ingestion. Furthermore, it has proved to be a safe natural compound in toxicity analysis which can be formulated into a new treatment option to cure multiple gastrointestinal ailments.

## Data Availability

The original contributions presented in the study are included in the article/Supplementary Material; further inquiries can be directed to the corresponding authors.

## Supplementary material

SUPPLEMENTARY TABLE S1 Binding energy (kcal/mol) and post-dock analysis of the best conformational pose of bergapten with adrenergic α1 receptor, muscarinic M1, muscarinic M3, dopaminergic D2, calmodulin, voltage-gated L-type calcium channel, histaminergic H1, H+/K+ ATPase pump, histaminergic H2, mu-opioid, and phosphodiesterase enzyme.

SUPPLEMENTARY TABLE S2 Pharmacokinetic and drug likeliness profiling of bergapten using SwissADME tool.
