# Supplementary material for: Pharmacological basis of bergapten in gastrointestinal diseases focusing on H+/K+ ATPase and voltage-gated calcium channel inhibition: A toxicological evaluation on vital organs
Source: Front Pharmacol. 2022 Nov 16;13:1005154. doi: 10.3389/fphar.2022.1005154 (PMC9709249; doi:10.3389/fphar.2022.1005154)
Supplement: Supplementary file 1 [file DataSheet4.docx]

**A**


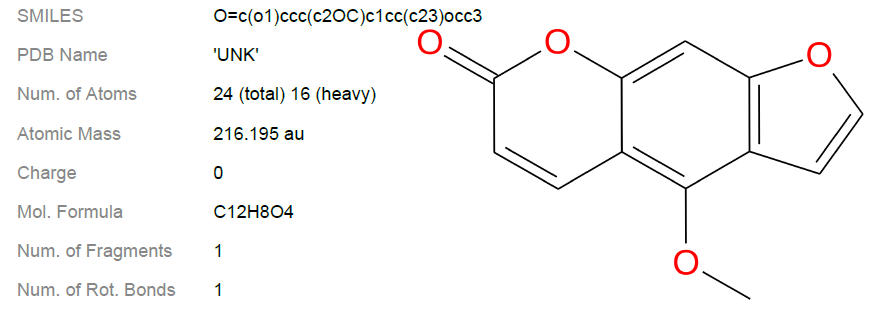


**B**

**Supplementary Figure S14**. Represent ligand (bergapten) information. **(A)** Represents root mean square deviation (RMSD) values, radius of gyrus (RG) value, intra molecular hydrogen bonds, molecular surface area (MSA), solvent accessible surface area (SASA), and polar surface area (PSA), respectively **(B)** represents structure of bergapten, number of atoms, atomic mass, charge, molecular formula, number of fragments and number of rotatable bonds.
