# Supplementary material for: Pharmacological basis of bergapten in gastrointestinal diseases focusing on H+/K+ ATPase and voltage-gated calcium channel inhibition: A toxicological evaluation on vital organs
Source: Front Pharmacol. 2022 Nov 16;13:1005154. doi: 10.3389/fphar.2022.1005154 (PMC9709249; doi:10.3389/fphar.2022.1005154)
Supplement: Supplementary file 2 [file DataSheet6.docx]

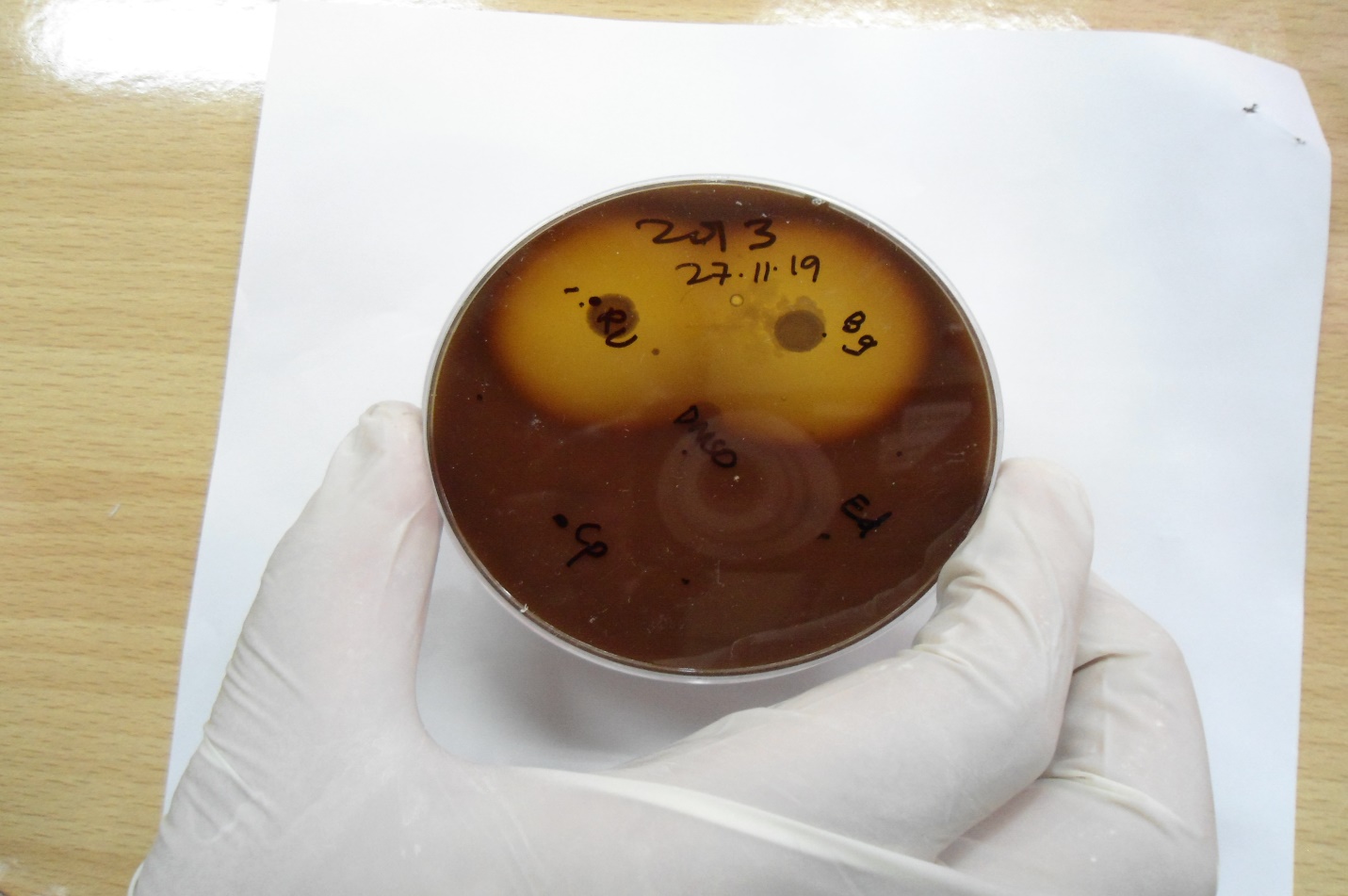


**Supplementary Figure S16:** Screening of bergapten against *H. pylori* resistant isolates.Effect of bergapten against *H. pylori* resistant isolates. Bg in figure indicates bergapten disc and marked zone of inhibition can be observed.
