# Supplementary material for: Pharmacological basis of bergapten in gastrointestinal diseases focusing on H+/K+ ATPase and voltage-gated calcium channel inhibition: A toxicological evaluation on vital organs
Source: Front Pharmacol. 2022 Nov 16;13:1005154. doi: 10.3389/fphar.2022.1005154 (PMC9709249; doi:10.3389/fphar.2022.1005154)
Supplement: Supplementary file 3 [file Table1.docx]

**Supplementary Table S1**. Binding energy (kcal/mol) and post-dock analysis of the best conformational pose of bergapten with adrenergic α_1_ receptor, muscarinic M_1_, muscarinic M_3_, dopaminergic D_2_, calmodulin, voltage gated L-Type calcium channel, histaminergic H_1,_ H^+^/K^+^ ATPase pump, histaminergic H_2,_ mu-opioid, and phosphodiesterase enzyme.

| **Target proteins** | **Bergapten** | | | | | | **Standard drugs** | | | | | | | |
| --- | --- | --- | --- | --- | --- | --- | --- | --- | --- | --- | --- | --- | --- | --- |
|  | **E-value (kcal/mol)** | **H bonds** | **Amino acids**  **forming H bonds** | **Pi-Pi bonds** | **Amino acids**  **forming**  **Pi-Pi bonds** | **Amino acids forming other hydrophobic interactions** | **Standard drugs** | **E-value (kcal/mol)** | **H bonds** | **Amino acids forming H bonds** | **Pi-Pi bonds** | **Amino acids**  **forming**  **Pi-Pi bonds** | **Amino acids forming other hydrophobic interactions** |  |
| Adrenergic α_1_ | -7.1 | 2 | TYR-B:316(2) | 2 | TYR-B:285, PHE-B:289 | ILE-B:178, ASP-B:106, VAL-B:107(2), CYS-B:110(2), SER-B:192 | Phenylephrine | -6.6 | 5 | THR 111 SER 192 CYS 110 ASP 106 TYR 316 | 1 | PHE 298 | VAL 107(2) CYS 110 PHE 312 |  |
| Muscarinic M_1_ | -7.2 | 2 | TYR 381, TYR 82 | 3 | TYR 404(3) | ILE 180, LEU 102 | Pirenzepine | -8.8 | 2 | THR 189 TYR 381 | 1 | TRP 400 | LEU 183 |  |
| muscarinic M_3_ | -5.6 | 0 | - | 1 | PHE 10 | PRO 13(2), THR 7(6), ASN 9(2), GLU 8(6) | Atropine | -5.6 | 2 | GLU 6, THR 7 | 1 | PHE 10 | ALA 5 GLU 8(11) PRO 13(2) ASN 9(2) THR 7(3) |  |
| Dopaminergic D_2_ | -6.3 | 2 | ASN-A:1002, TRP-A:1158 | 0 | - | VAL-A:1094(3), LYS-A:369(2), ASP-A:1092, ARG-A:220 | Domperidone | -9.3 | 3 | SER 193 TYR 408 TRP 413 | 5 | PHE 389 PHE 189 HIS 393(2) TRP 100 | VAL 115 VAL 190 ILE 184 PHE 189 TYR 408 TYR 416 VAL 91 LEU 94 |  |
| Calmodulin | -5.7 | 0 | - | 0 | - | ILE 125, VAL 136, MET 144(2), LEU 105(2) | Calmidazolium | -8.2 | 0 | - | 0 | - | LEU 105(3), VAL 136, ILE 100, ILE 125, PHE 92, VAL 108, MET 124(2), LEU 39(2), LEU 116, LEU 112(2), MET 109, LEU 18, VAL 35, PHE 19, ALA 15 |  |
| Calcium channel | -6.3 | 1 | ARG-A:228 | 0 | - | VAL-A:339, PRO-A:337, ASP-A:384(3), ARG-A:228 | Verapamil | -6.7 | 1 | SER 334 | 2 | PHE 308 PHE 63 | PHE 63 ARG 312 VAL 223(3) PHE 308 ALA 62 |  |
| Histaminergic H1 | -6.3 | 1 | TRP 93 | 0 | - | ARG 175, ASP 183, ARG 97, CYS 100, LEU 101, CYS 180(2), | Pyrilamine | -6.3 | 2 | GLY 1030 GLU 1011 | 1 | PHE 1104 | LEU 1032 GLY 1030 ALA 1073 ALA 1074 HIS 1031 |  |
| H+/K+ ATPase | -8.1 | 1 | THR 880 | 0 | - | VAL 1000(3), LEU 62(3), PRO 1003, CYS 58 | Omeprazole | -8.1 | 0 | - | 2 | TYR 799(2) | CYR 213(2) ALA 335(2) VAL 331(3) ALA 123(2) GLN 127 ILE 126 ASP 132 |  |
| Histaminergic H2 | -5.7 | 3 | TYR 278, TYR 94, SER 75 | 2 | TYR 78(2) | ALA 271(2), LEU 274, GLU 270 | Ranitine | -5.7 | 4 | SER 75(2) TYR 94 TYR 278 | 1 | TYR 78 | ALA 271 LYS 175 GLU 267 SER 75(2) GLN 79 TYR 78 |  |
| Mu-opioid | -9.5 | 2 | SER 189, THR 102 | 0 | - | THR 274, CYS 148(2), CYS 317(2) | loperamide | -9.5 | 1 | ARG 150 | 0 | - | VAL 320 HIS 62 CYS 317 CYS 148 |  |
| Phosphodiesterase enzyme | -7.4 | 2 | ASN 405, TYR 424 | 4 | PHE 456(3), PHE 251 | LEU 420(3), ILE 403 | Papaverine | -8.1 | 2 | GLU 453 TYR 424 | 2 | PHE 456(2) | ASP 402 LEU 420(2) TYR 424 HIS 296 |  |
