# Supplementary material for: Pharmacological basis of bergapten in gastrointestinal diseases focusing on H+/K+ ATPase and voltage-gated calcium channel inhibition: A toxicological evaluation on vital organs
Source: Front Pharmacol. 2022 Nov 16;13:1005154. doi: 10.3389/fphar.2022.1005154 (PMC9709249; doi:10.3389/fphar.2022.1005154)
Supplement: Supplementary file 4 [file DataSheet3.docx]

**A**

**B**

**C**

**Supplementary figure S13. Properties of target protein Voltage gated L-Type Calcium channels PDBID: 1T3S**

Part (**A**) represents the total residues, protein chain, atoms, heavy atoms, and charged atoms. Part (**B**) shows the mean root square fluctuation (RMSF) in angstrom (Å) of the target protein. Part (**C**) represents the protein secondary structure elements (SSE), alpha-helices shown in red, and beta-strands shown in blue monitored through simulation. The plot summarizes the SSE composition for each trajectory frame over the course of simulation.
