# Supplementary material for: Pharmacological basis of bergapten in gastrointestinal diseases focusing on H+/K+ ATPase and voltage-gated calcium channel inhibition: A toxicological evaluation on vital organs
Source: Front Pharmacol. 2022 Nov 16;13:1005154. doi: 10.3389/fphar.2022.1005154 (PMC9709249; doi:10.3389/fphar.2022.1005154)
Supplement: Supplementary file 5 [file DataSheet5.docx]

**A**

**B**

**Supplementary Figure S15**. **(A)** indicates the root mean square fluctuation (RMSF) of the atomic positions for the bergapten on 4UX2 **(B)** indicates RMSF of the atomic positions for bergapten on 1T3S.
