# Supplementary material for: Pharmacological basis of bergapten in gastrointestinal diseases focusing on H+/K+ ATPase and voltage-gated calcium channel inhibition: A toxicological evaluation on vital organs
Source: Front Pharmacol. 2022 Nov 16;13:1005154. doi: 10.3389/fphar.2022.1005154 (PMC9709249; doi:10.3389/fphar.2022.1005154)
Supplement: Supplementary file 6 [file Table2.docx]

**Supplementary Table S2.** Pharmacokinetic and drug likeliness profiling of bergapten using SwissADME tool.


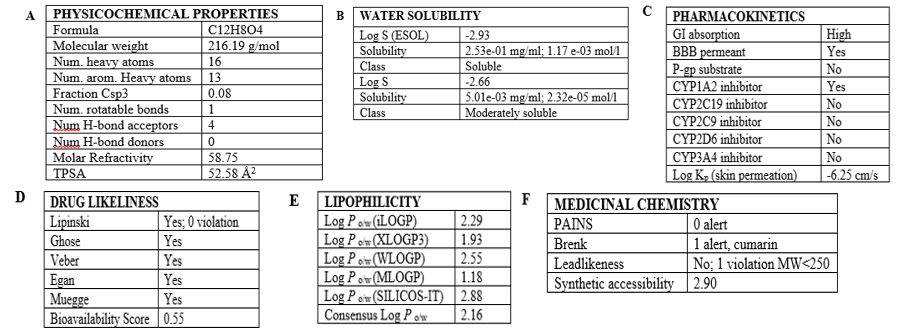
**A**: represents physicochemical properties of bergapten **B:** represents water solubility, **C:** represents pharmacokinetics **D:** represents druglikeliness **E:** represents lipophilic profile **F:** reperesents medicinal likeability of bergapten.
