# Supplementary material for: Pharmacological basis of bergapten in gastrointestinal diseases focusing on H+/K+ ATPase and voltage-gated calcium channel inhibition: A toxicological evaluation on vital organs
Source: Front Pharmacol. 2022 Nov 16;13:1005154. doi: 10.3389/fphar.2022.1005154 (PMC9709249; doi:10.3389/fphar.2022.1005154)
Supplement: Supplementary file 8 [file DataSheet1.docx]

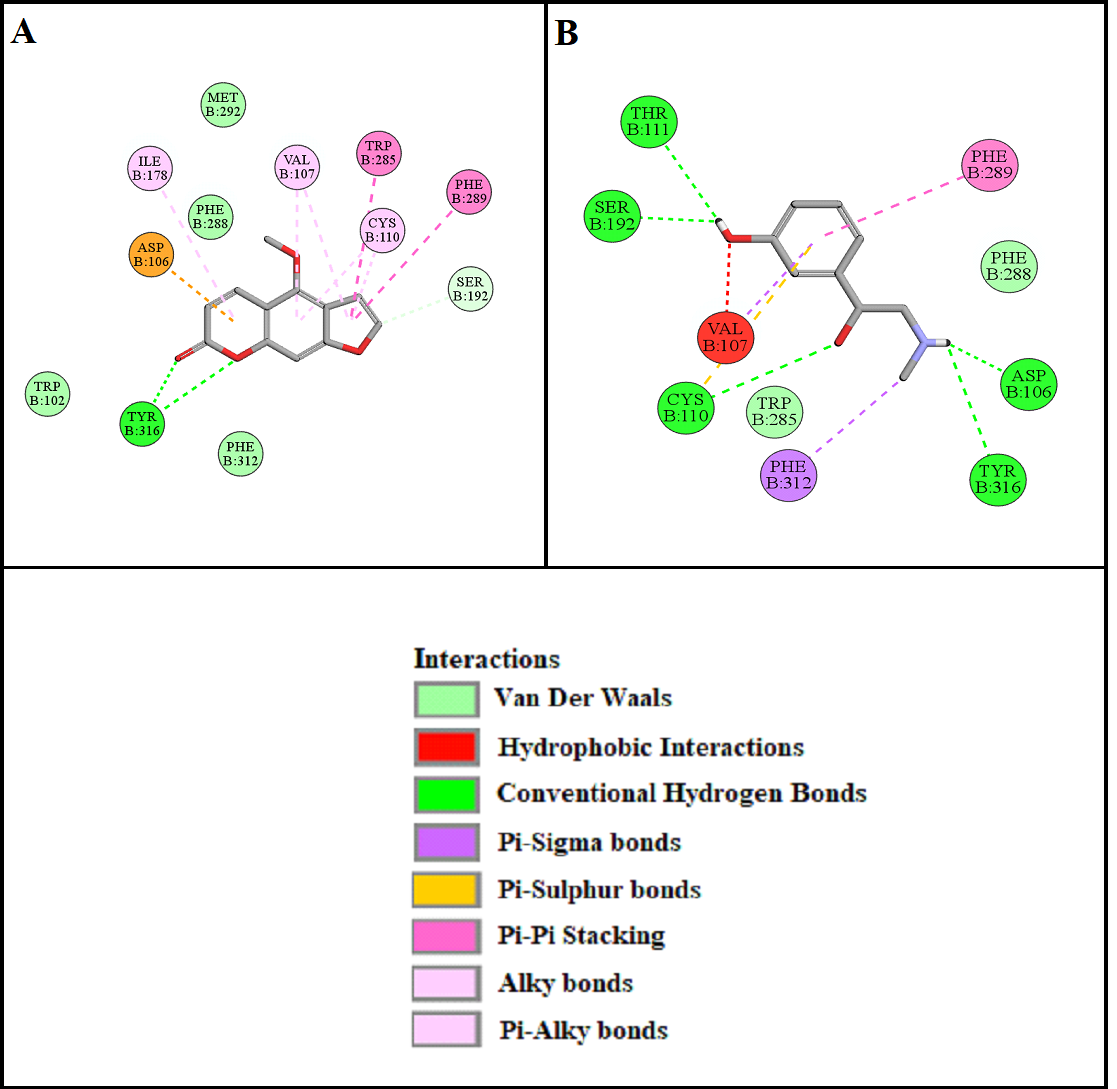


**Supplementary Figure S1: A** and **B** and represents 2D interactions of bergapten and phenylephrine with alpha adrenergic receptor (α_1_) respectively, drawn through Biovia Discovery Studio Visualizer 2016.


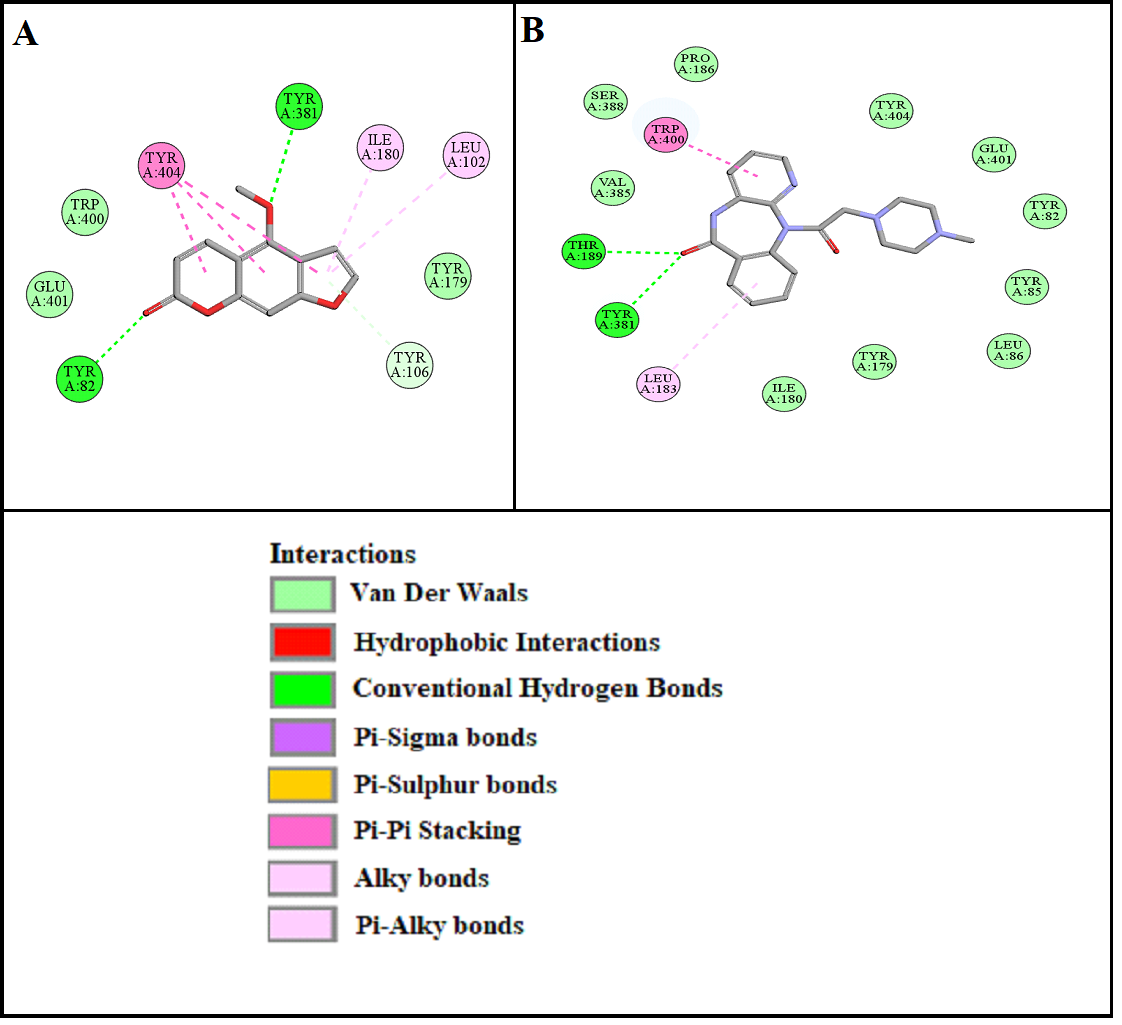


**Supplementary Figure S2: A** and **B** represents interactions of bergapten and pirenzepine with muscarinic M_1_ receptor (M_1_) respectively, drawn through Biovia Discovery Studio Visualizer 2016.


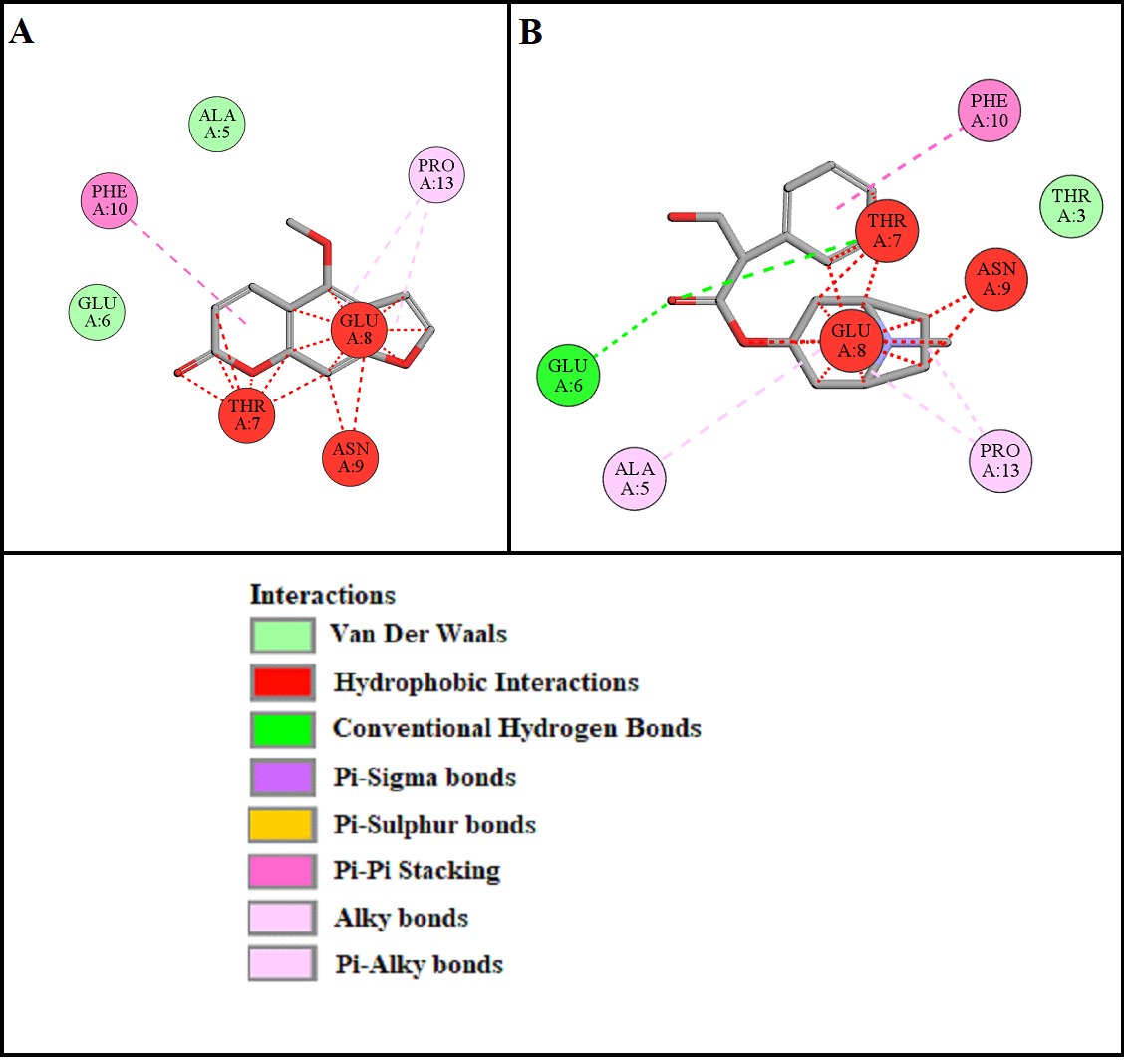


**Supplementary Figure S3: A** and **B** represents 2D interactions of bergapten and atropine with muscarinic receptor (M_3_) respectively, drawn through Biovia Discovery Studio Visualizer 2016.


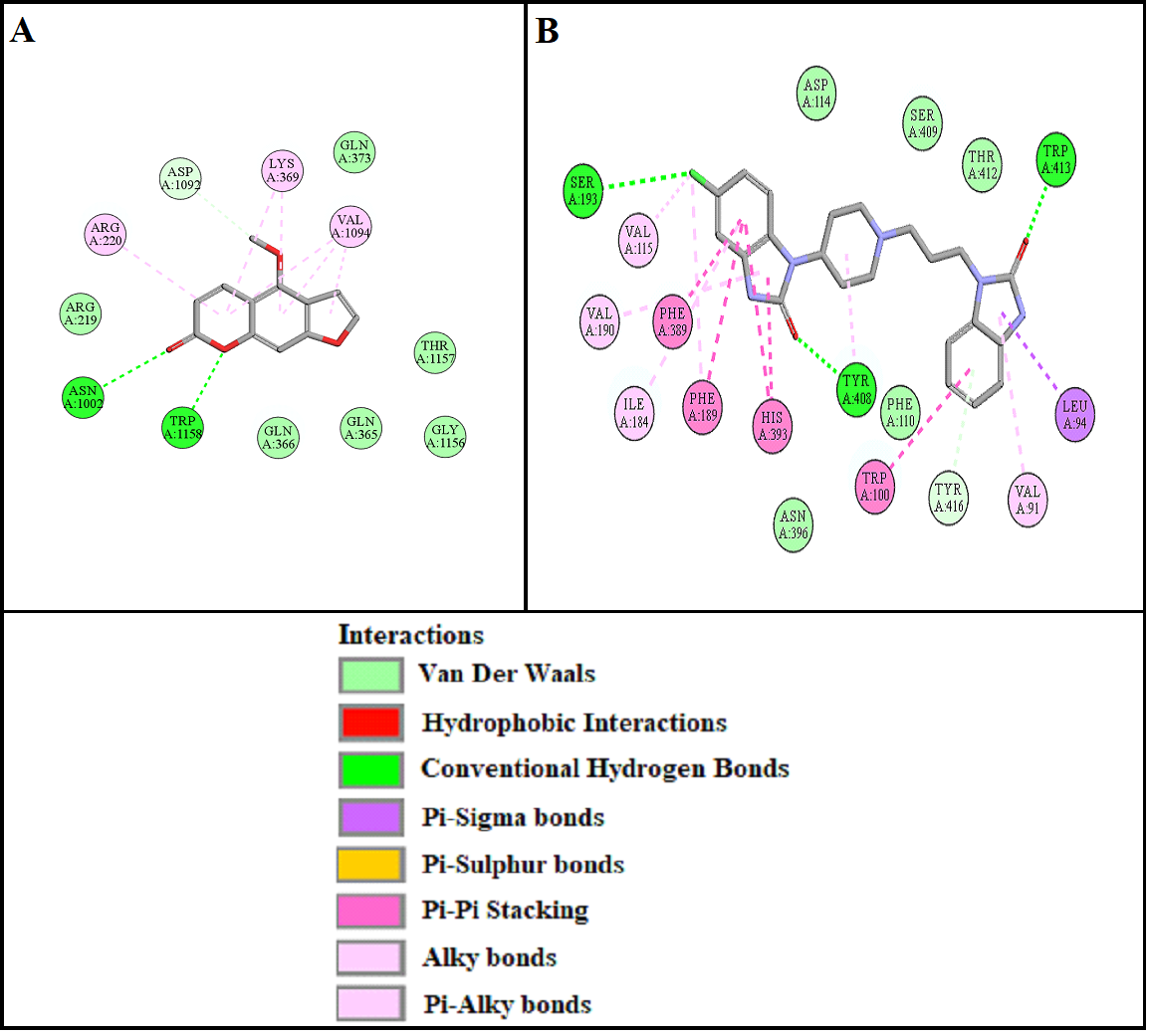


**Supplementary Figure S4. A** and **B** represents 2D interactions of bergapten and domperidone with dopaminergic receptor (D_2_) respectively, drawn through Biovia Discovery Studio Visualizer 2016.


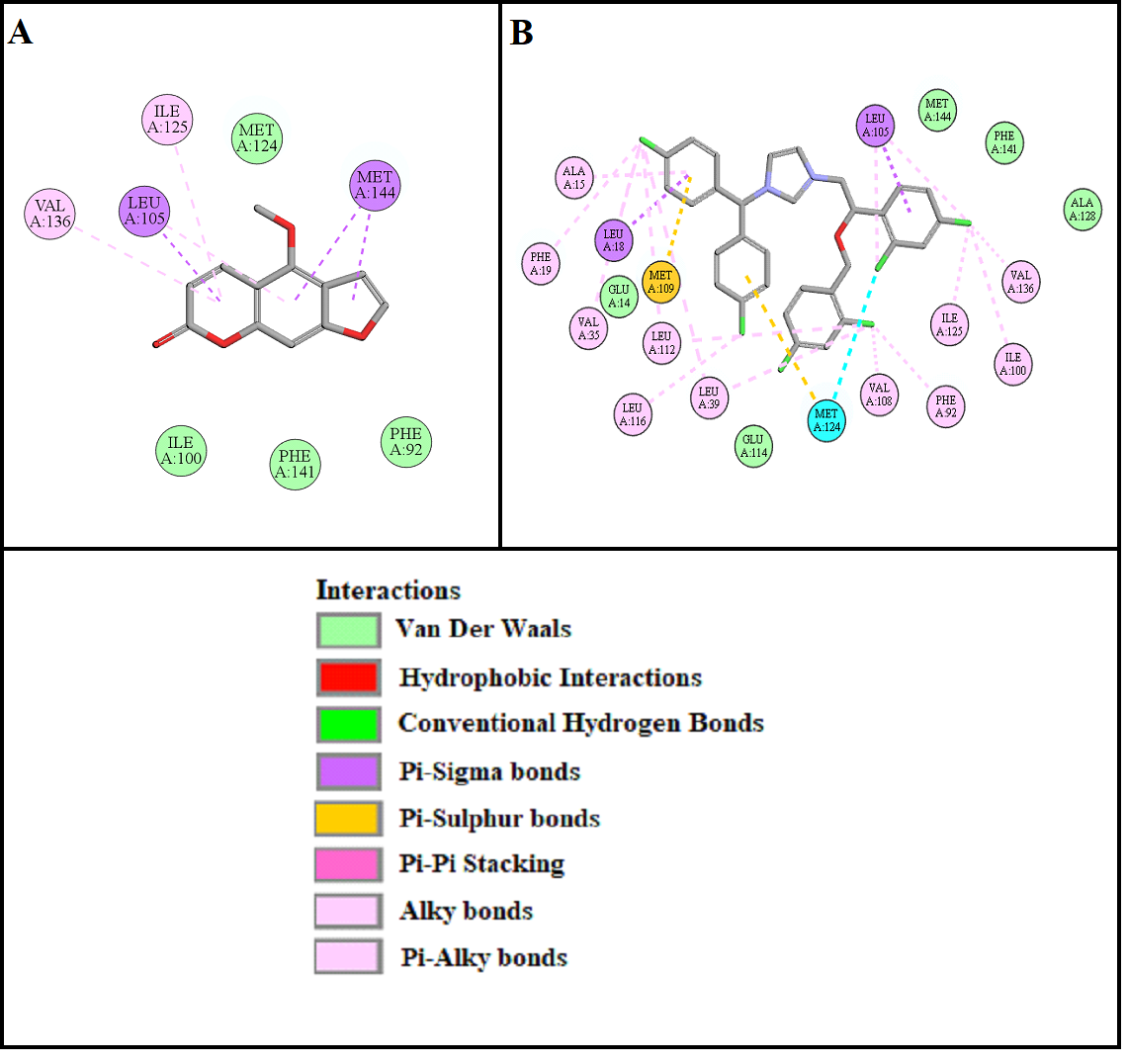


**Supplementary Figure S5: A** and **B** represents 2D interactions of bergapten and calmidazolium with calmodulin respectively, drawn through Biovia Discovery Studio Visualizer 2016.


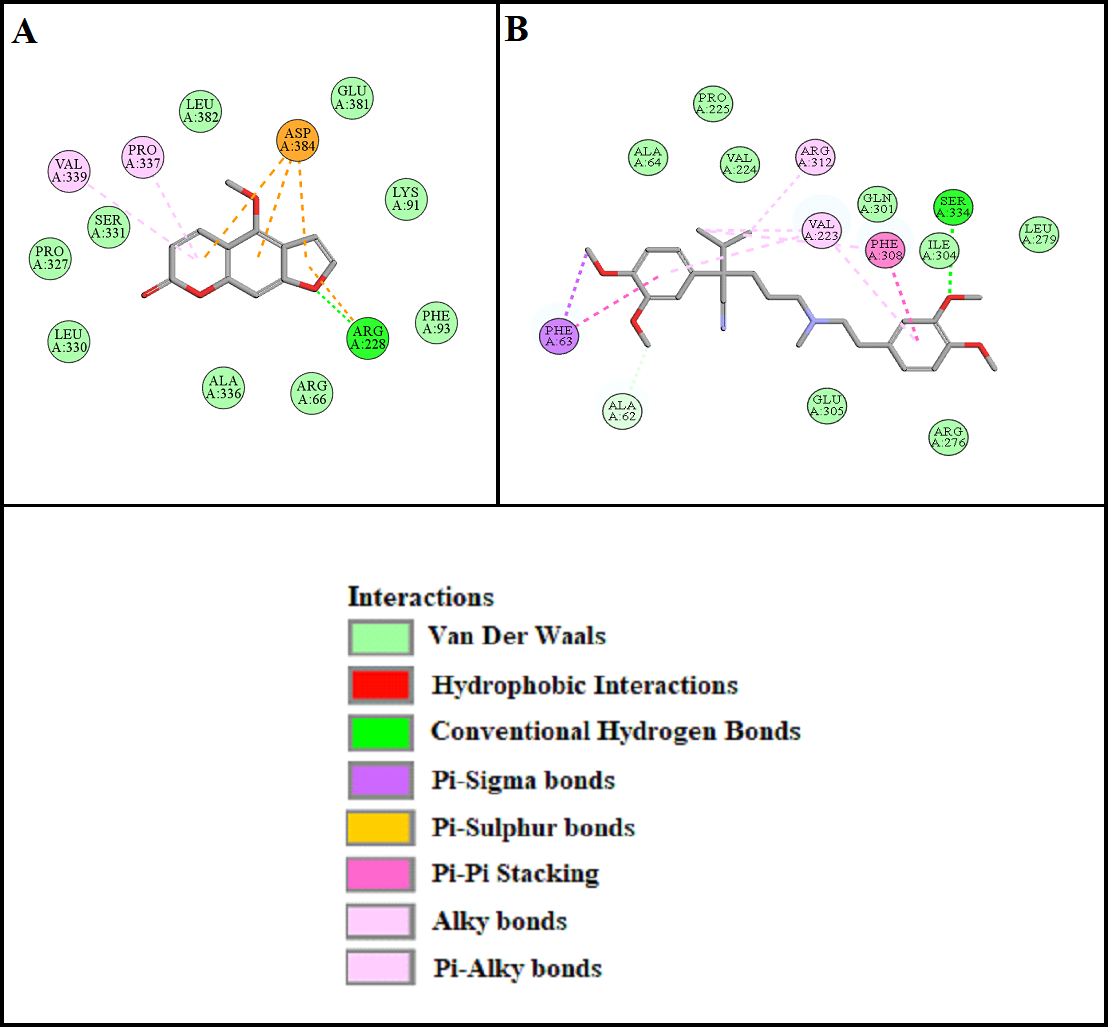


**Supplementary Figure S6. A** and **B** represents **2D** interactions of bergapten and verapamil with voltage gated L-Type calcium channel respectively, drawn through Biovia Discovery Studio Visualizer 2016.


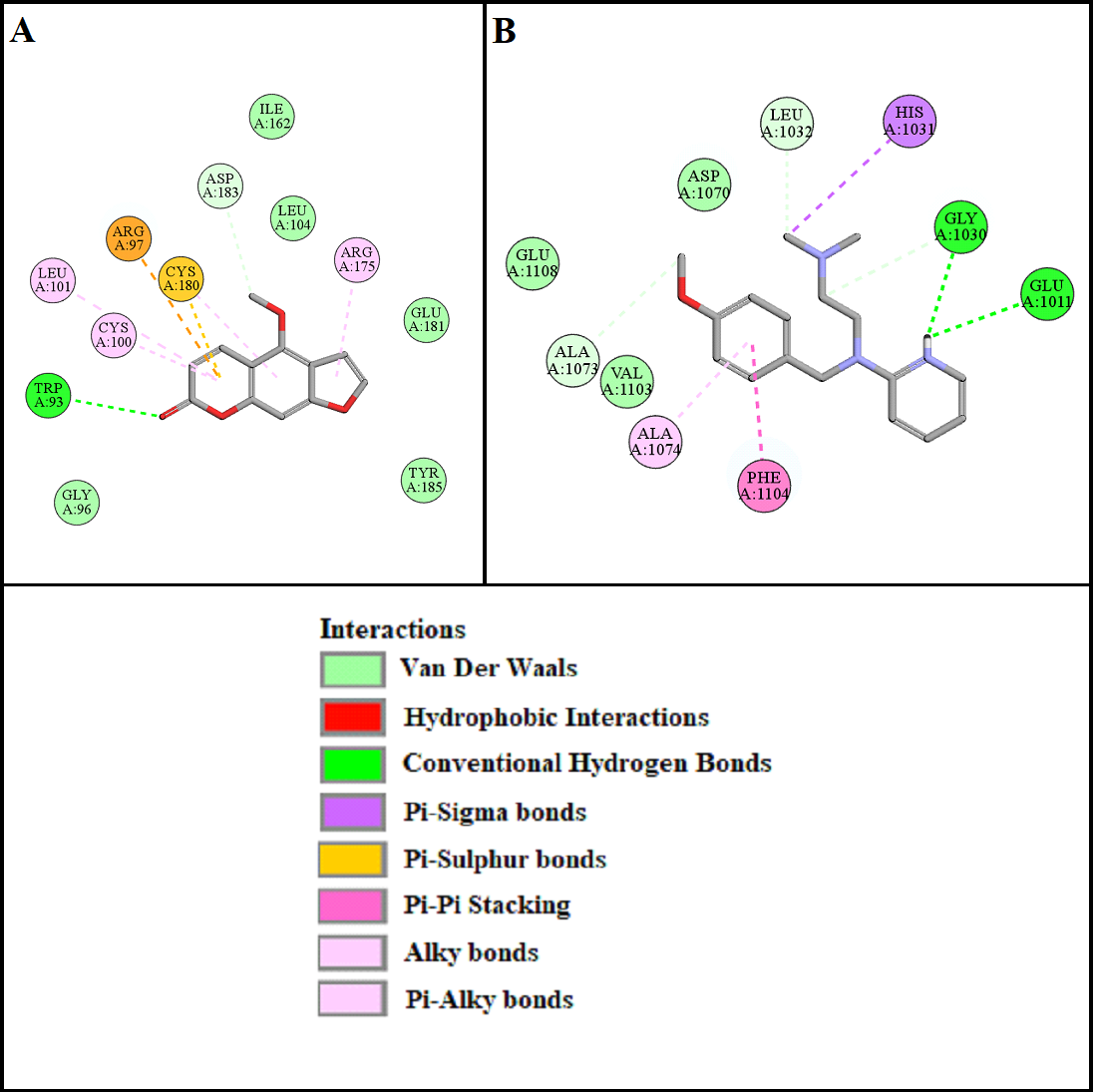


**Supplementary Figure S7. A** and **B** demonstrate **2D** interactions of bergapten and pyrilamine with histaminergic receptor (H_1_) respectively, drawn through Biovia Discovery Studio Visualizer 2016.


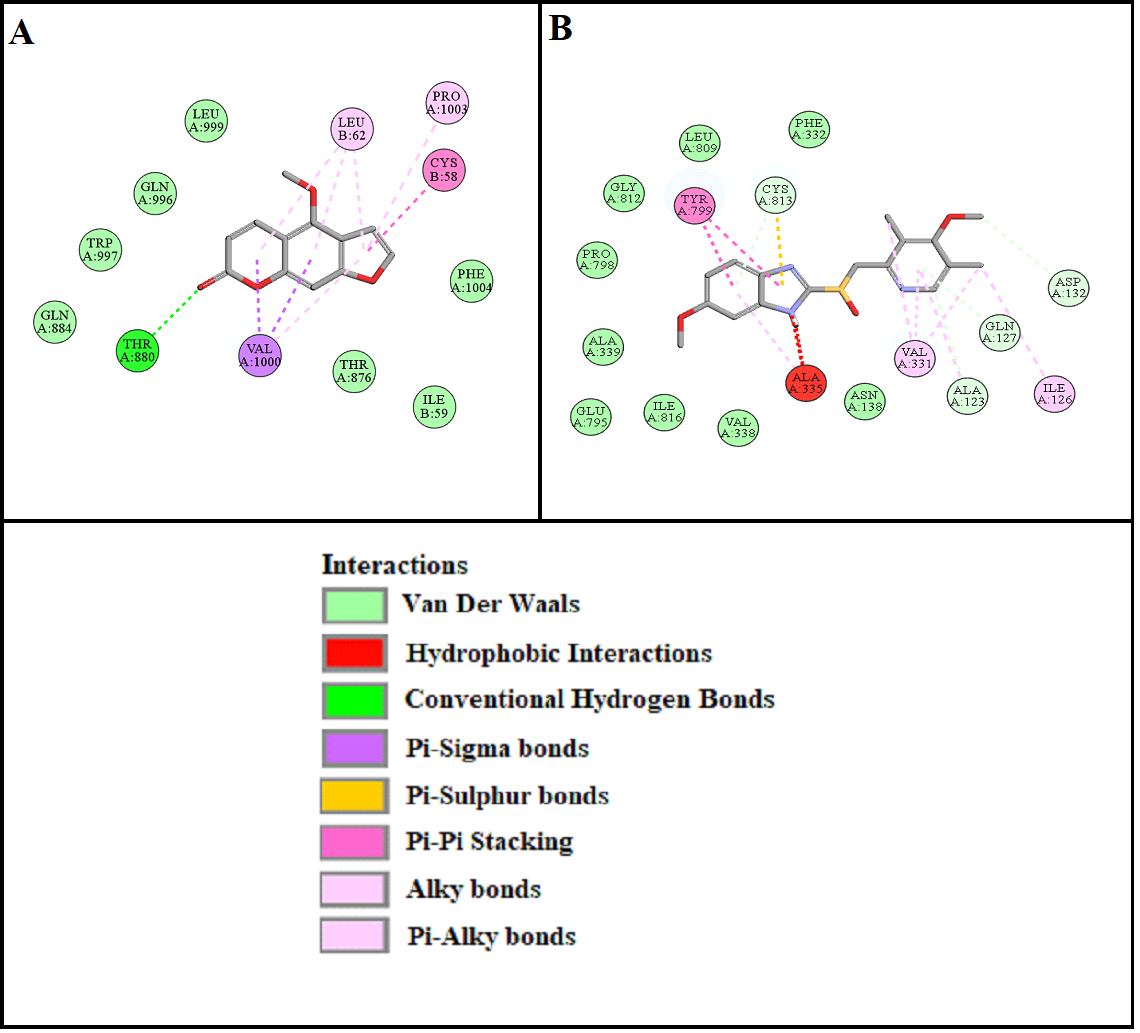


**Supplementary Figure S8. A** and **B** demonstrate **2D** interactions of bergapten and omeprazole with hydrogen potassium ATPase, also known as H^+^/K^+^ ATPase receptor respectively, drawn through Biovia Discovery Studio Visualizer 2016.


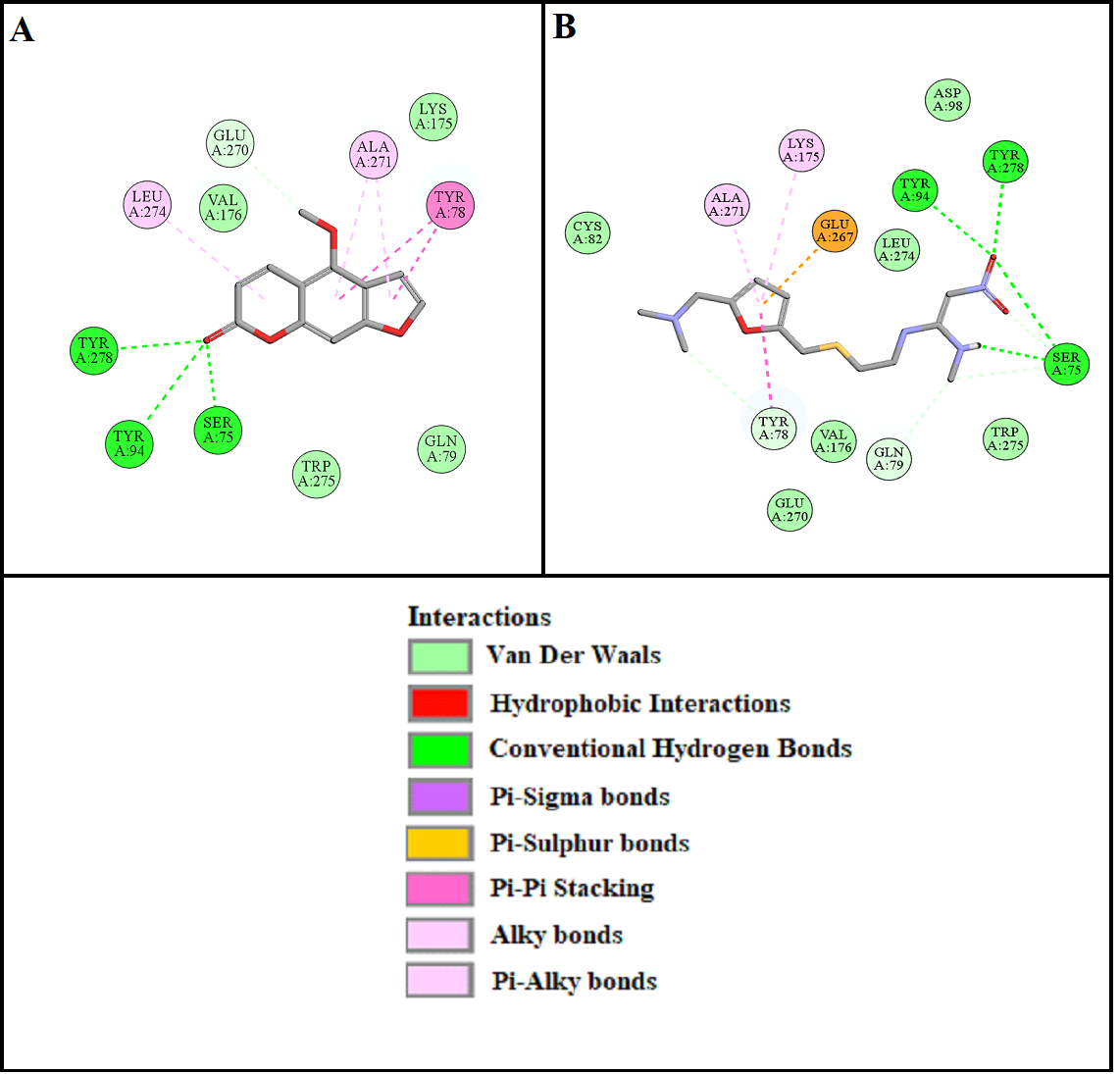


**Supplementary Figure S9. A** and **B** demonstrate **2D** interactions of bergapten and ranitidine with histaminergic receptor (H_2_) respectively, drawn through Biovia Discovery Studio Visualizer 2016.


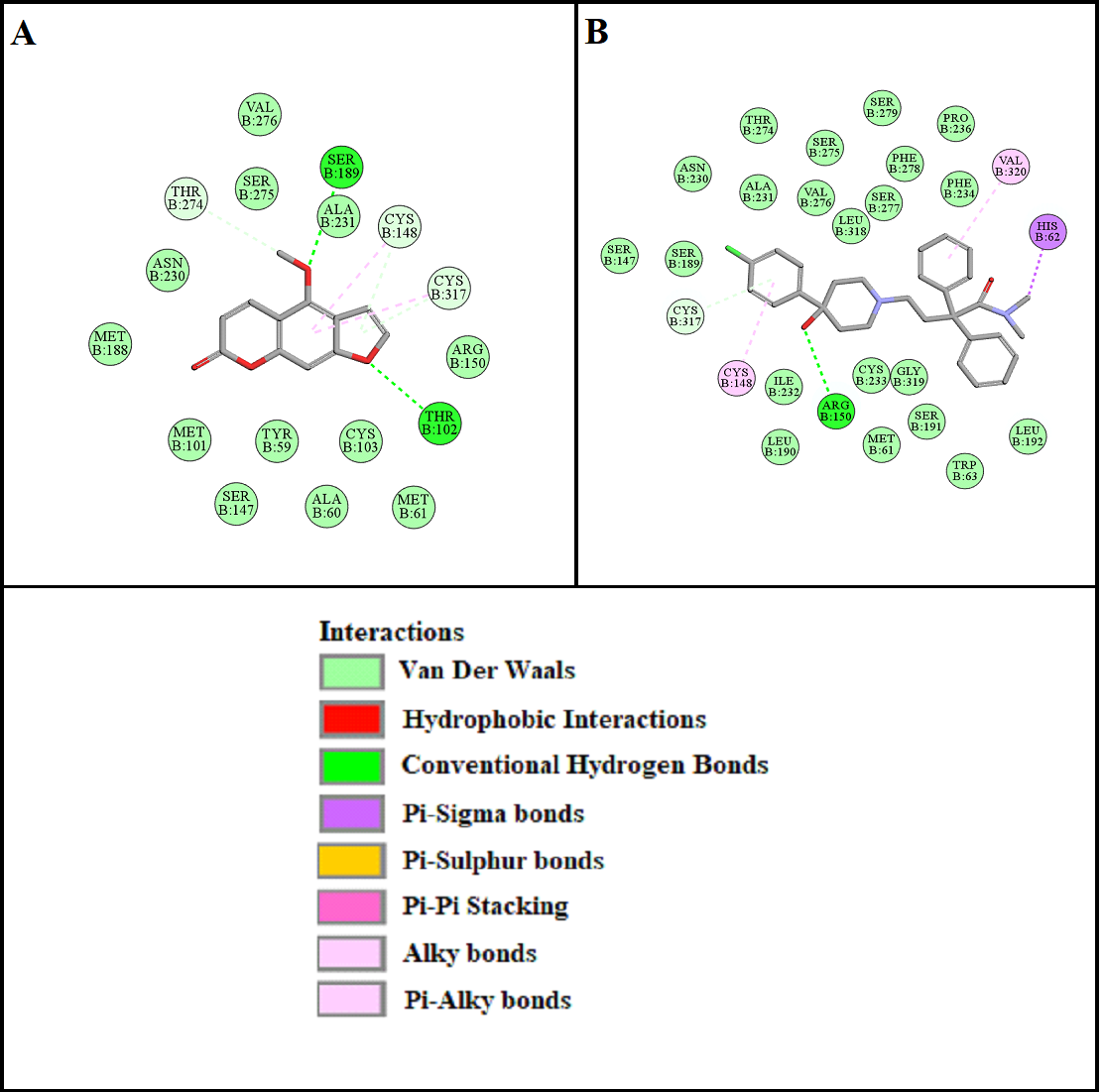


**Supplementary Figure S10. A** and **B** demonstrate **2D** interactions of bergapten and loperamide with mu-opioid receptor (μ) respectively, drawn through Biovia Discovery Studio Visualizer 2016.

**
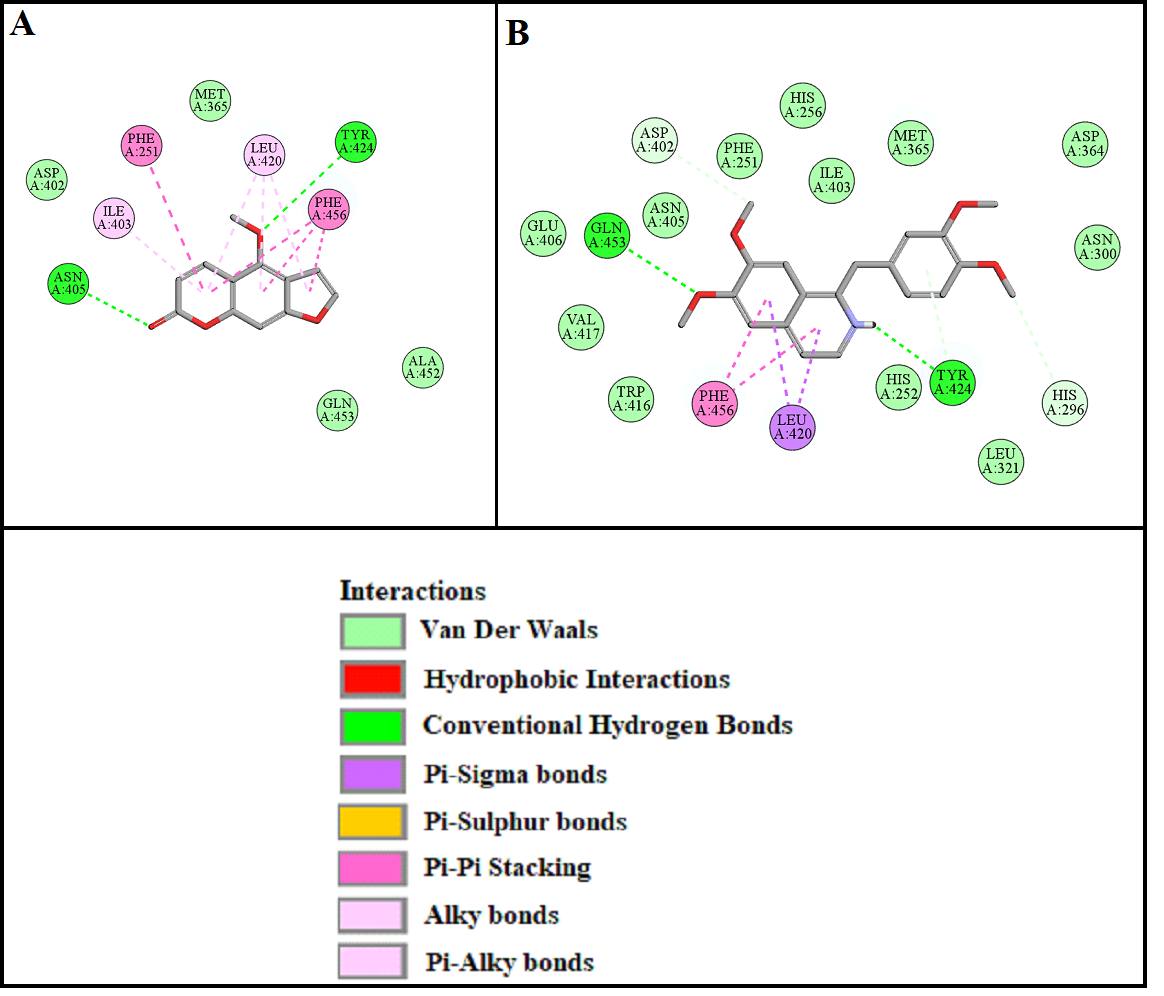
**

**Supplementary Figure S11. A** and **B** demonstrate **2D** interactions of bergapten and papaverine with phosphodiesterase enzyme respectively, drawn through Biovia Discovery Studio Visualizer 2016.
